# Chinese clinical practice guidelines for super minimally invasive surgery of digestive tract tumors

**DOI:** 10.1515/jtim-2025-0095

**Published:** 2025-12-22

**Authors:** Qianqian Chen, Yaoqian Yuan, Huikai Li, Yutong Sun, Xiaoliang Zhu, Yaqi Zhai, Miao Liu, Kunming Lyu, Bo Ning, Qun Shao, Junfeng Xu, Zhengcong Zhang, Yunqi Li, Shuai Tian, Xinye Zuo, Ke Han, Jiafeng Wang, Chen Du, Lei Zhang, Jiajun Du, Liangying Liu, Xin Chen, Enqiang Linghu, Chinese Society of Digestive Endoscopy, SMIS Committee of World Endoscopy Organization

**Affiliations:** Department of Gastroenterology, the First Medical Center of Chinese People's Liberation Army General Hospital, Beijing, China; The First Hospital of Lanzhou University, Lanzhou, Gansu Province, China; Department of Epidemiology, Chinese People's Liberation Army Medical School, Chinese People's Liberation Army General Hospital, Beijing, China; Department of Gastroenterology, the Second Medical Center of Chinese People's Liberation Army General Hospital, Beijing, China; Library of the Graduate School, Chinese People's Liberation Army General Hospital, Beijing, China

**Keywords:** super minimally invasive surgery, esophageal cancer, gastric cancer, colon cancer, rectal cancer, precancerous lesions, guidelines

## Abstract

The mode of organ resection and reconstruction that has been used to treat digestive tract tumors (DTTs) can cure the disease. However, it involves the surgical resection of critical structures (such as the cardia, pylorus, and anus) and gastrointestinal reconstruction, which alter the physiological anatomy of the digestive system. These changes often lead to numerous postoperative complications and severely affect the patient’s quality of life (*e.g*., refractory gastroesophageal reflux following proximal gastrectomy, dumping syndrome after subtotal gastrectomy, loss of anal function after low rectal surgery). For the defect of this mode, professor Linghu Enqiang proposed the new mode that was “curing the disease and restoring normal function” in 2016, which was named as this new mode: Super Minimally Invasive Surgery (SMIS). To accomplish various types of SMIS, four operative channels were developed: the natural cavity channel, the tunnel channel, the puncture channel, and the multi-cavity channel. SMIS, with its advantages of minimal trauma and organ function preservation, has been recognized by authoritative domestic and international organizations and has developed rapidly. Based on its clinical value and the need for wider application, there is an urgent need to establish standardized guidelines to guide practice. This guideline was developed by leading organizations such as the SMIS committee of World Endoscopy Organization (WEO) and Chinese Society of Digestive Endoscopy (CSDE), in collaboration with multidisciplinary experts from gastroenterology, general surgery, and pathology. Systematic searches were conducted in nine major databases, including PubMed, Embase, and China National Knowledge Infrastructure (CNKI), for both Chinese and English literature published before 2025. Evidence from randomized controlled trials (RCTs), observational studies, and case series was included, with the quality of evidence and recommendation strength evaluated using the GRADE system (high-level evidence:RCTs; low-level evidence: observational studies). The recommendations were refined through several rounds of expert discussions and voting, and were reported following the AGREE II and RIGHT reporting standards. This guideline has been registered on the PREPARE (Practice Guideline REgistration for transPAREncy; registration number: PREPARE-2024CN1183). This guideline addresses 15 issues related to SMIS treatment for esophageal cancer (EC), gastric cancer (GC), colorectal cancer (CRC), their corresponding precancerous lesions, and precancerous lesions of the duodenal papilla. It provides corresponding recommendations in three main areas: (1) Definitions and principles: SMIS should meet ten core criteria, including organ preservation, complete resection (R0), and sterile procedures. It also standardizes naming conventions (*e.g*., “Super minimally invasive non-full-thickness resection of lower esophageal squamous carcinoma *via* the oral cavity”). (2) Surgical recommendations: EC: For early and precancerous lesions, SMIS of non-full-thickness resection (non-FTR) is preferred. For circumferential involvement ≥ 1/2, SMIS *via* tunnel channel for non-FTR is recommended. If the wound circumference is ≥ 75%, the use of corticosteroids or stents to prevent stenosis is advised. GC: For T1a-T1b stage and precancerous lesions, SMIS non-FTR or full-thickness resection (FTR) is preferred, with individualized plans based on the risk of lymph node metastasis (LNM). CRC: SMIS of non-FTR or FTR is recommended as the first-line treatment for T1a-T1b stage and precancerous lesions. For locally advanced rectal cancer (LARC) that achieves clinical remission after neoadjuvant therapy, SMIS of FTR can be considered to assess pathological remission. Duodenal papilla precancerous lesions: SMIS resection via the oral cavity is preferred. Postoperatively, whether to add pancreaticoduodenectomy and follow-up strategies should be determined based on pathology. (3) Postoperative management: A SMIS treatment cure evaluation system for early gastric cancer (EGC) was established, divided into SMIS-Cure A(cured), SMIS-Cure B (clinically cured), and SMIS-Cure C (surgical reassessment), which guides follow-up. For CRC or precancerous lesions, R0 resection is the standard for cure, and follow-up plans are developed according to risk stratification. This guideline systematically integrates the evidence from SMIS in the treatment of DTTs with expert consensus, establishing a standardized pathway centered on organ function preservation. It shifts the treatment model from “cure first” to “cure-function balance”. Its application is expected to reduce overtreatment, improve the patient’s quality of life, and provide a framework for future technological iterations and the expansion of indications. It should be continuously optimized with multicenter clinical data and long-term follow-up results to achieve more precise, individualized treatment.

## Background

Digestive tract tumors (DTTs) represent a major clinical challenge due to their high incidence and mortality, as well as the substantial physical and psychological burden associated with surgical treatment. At present, curative resection remains the standard and preferred approach for the radical treatment of DTTs. However, surgical strategies based on organ removal and anatomical reconstruction inevitably lead to significant short-term complications and long-term impairments in quality of life. For example, total gastrectomy often results in nutrient malabsorption, leading to anemia and osteoporosis; patients undergoing subtotal gastrectomy may suffer from postural intolerance (inability to lie flat), diarrhea, gastroparesis, and dumping syndrome. Esophageal or colorectal resections are frequently associated with anastomotic leakage and anastomotic stricture, while rectal surgery often necessitates loss of anal preservation. Similarly, pancreaticoduodenectomy (Whipple procedure) alters the physiological route of food digestion, and may induce complications such as biliary fistula and pancreatic fistula^[1]^. Notably, organ resection and anatomical reconstruction also pose potential drawbacks. A study on post-gastrectomy patients revealed that the surgery significantly affects the activity of the posterior cingulate cortex, suggesting an impact beyond local anatomical changes^[2]^. In contrast, SMIS, organ-preserving curative procedures, which maintain the integrity of normal anatomical structures, can effectively avoid these complications. By achieving radical disease eradication while preserving organ function, this approach represents a new paradigm in surgical oncology.

The Super Minimally Invasive Surgery (SMIS) paradigm was first proposed by professor Enqiang Linghu in 2016 as a revolutionary surgical concept that redefines traditional approaches based on organ resection and anatomical reconstruction^[3]^. This innovative model utilizes four principal access channels—*via* the natural cavity channel, the tunnel channel, the puncture channel, and the multi-cavity channel, and encompasses dozens of surgical techniques and nearly a hundred procedural variations, all aimed at achieving the ultimate goal of “curing disease while restoring the organ to its original integrity”^[4]^. Guided by the philosophy of SMIS, therapeutic interventions strive for near-perfect outcomes, enabling patients to retain their preoperative quality of life and ensuring that survival remains unaffected by the disease itself. The SMIS paradigm promotes a treatment philosophy grounded in the body’s natural anatomical order, emphasizing organ preservation, functional restoration, and minimal disruption to physiological structures. Moreover, the underlying principles of SMIS are universally applicable across all domains of invasive medical therapy, representing the ultimate goal and future direction of surgical innovation and practice.

Since it was first proposed, the concept of SMIS has received official recognition from both national and international academic organizations. In 2019, the SMIS Collaborative Group was established under the Chinese Society of Digestive Endoscopy (CSDE). In 2021, the World Endoscopy Organization (WEO) founded the SMIS Committee, and in the same year, the National Committee for Terms in Sciences and Technologies of China officially included SMIS in the Terminology of Digestive Endoscopy, thereby standardizing its nomenclature within the professional community^[5]^. In 2022, the 14th Five-Year National Key R&D Program funded the “Establishment and Application Model Research of the SMIS Efficacy Evaluation System for Digestive Tract Tumors.” Building on these milestones, SMIS has played an increasingly important role in the treatment of DTTs, showing rapid progress and widespread clinical adoption worldwide (Table 1, 2). Consequently, the development of a scientific and standardized clinical practice guideline has become both necessary and urgent. This guideline aims to provide evidence-based and expert consensus-driven recommendations for the clinical application of SMIS in the management of esophageal, gastric, colorectal, and duodenal tumors, through a systematic evaluation of current literature and multidisciplinary expert collaboration.

**Table 1:** Terminology and definitions used in these guidelines

Terminology	Definition
Super minimally invasive surgery (SMIS)	A general category of surgeries aimed at removing diseased tissue that requires surgical excision or incision, while ensuring that the anatomical integrity of the organ remains unchanged, thus achieving the goal of complete disease removal.
Early cancer (EC)	Malignant tumors confined to the mucosa and/or submucosa, without deeper invasion, regardless of whether lymph node metastasis is present.
En-bloc resection	Removal of the entire tumor, heterogeneous hyperplasia, and/or cancerous tissue in one piece, rather than in multiple pieces.
R0 resection	Resection margins are free of disease, without cancerous tissue present at the edges of the excised tissue.
Curative resection (CR)	Histological evaluation of the excised specimen meets the following standards. 1. Pathological assessment indicates that both the horizontal and vertical margins are free of malignant cells.
	2. Generally, the tumor has well-differentiated cellular characteristics.
	3. No lymphatic, vascular, or nerve invasion is present.
	4. The tumor has not invaded beyond the submucosa.
	5. A more detailed determination is provided for the curative evaluation of different types of digestive tract tumors.
Cancer recurrence	Pathological confirmation of recurrence at a previously resected or surgical site, or lymph node metastasis.
Clinical complete response	Following evaluation with high-definition endoscopy (combined with enhanced imaging techniques such as endoscopic ultrasound and narrow-band imaging when necessary), the mucosa at the original tumor site has returned to normal or shows only scarring and deformation, with no clear residual tumor lesions observed.
Depth of invasion	M1: *In situ* carcinoma, non-invasive carcinoma confined to the epithelium.
	M2: Microinvasive carcinoma invading the lamina propria.
	M3: Microinvasive carcinoma extending into the muscularis mucosae.
	SM1: Microinvasive carcinoma invading the upper third of the submucosa (esophagus ≤ 200 μm; stomach ≤ 500 μm; colon ≤ 1000 μm).
	SM2: Microinvasive carcinoma invading the middle third of the submucosa (esophagus 200–400 μm; stomach 500–1000 μm; colon 1000–2000 μm).
	SM3: Microinvasive carcinoma invading the lower third of the submucosa (esophagus 400–600 μm; stomach 1000–1500 μm; colon 2000–3000 μm).
Dysplasia	Lesions that exhibit clear tumor-like changes histologically but lack evidence of tissue invasion. This term differs from intraepithelial neoplasia in that dysplasia refers to lesions with morphological features characteristic of tumors. It is classified into mild dysplasia, moderate dysplasia, and severe dysplasia.
Intraepithelial neoplasia	A range of diseases in which “cells or structures undergo changes,“ and is believed to reflect underlying molecular biological abnormalities that may lead to the progression of invasive cancer. It is classified into high grade intraepithelial neoplasia (HIN) and low grade intraepithelial neoplasia (LIN).
Near clinical complete response	Following MRI evaluation, only extremely subtle residual signs were observed at the original tumor site, making it impossible to definitively distinguish whether these represent residual tumor or post-treatment inflammatory/fibrotic reaction
Pathologic complete response	Following comprehensive pathological examination of the resected specimen, no viable tumor cells were detected in either the primary tumor site or regional lymph nodes. In DTTs, the original tumor location may reveal only extensive fibrous tissue, mucus pools, necrotic material, or inflammatory cell infiltration, but no living tumor cells.
Precancerous lesion (PL)	A type of pathological histological change prone to malignant transformation, including dysplasia and intraepithelial neoplasia.
Ulcerative lesion (UL)	Lesions occurring in the mucosa and/or submucosa, specifically manifested as disruption of mucosal integrity with visible loss or exposure of the basement membrane and submucosa (or extending into the submucosa), forming a depressed defect. This is often accompanied by inflammatory response, necrotic tissue, possible exudate, hemorrhage, and scarring.

**Table 2:** Definition of SMIS and the ten principles for its implementation

Definition of Super minimally invasive surgery and the ten principles for its implementation	
Definition	A general category of surgeries aimed at removing diseased tissue that requires surgical excision or incision, while ensuring that the anatomical integrity of the organ remains unchanged, thus achieving the goal of complete disease removal.
Principles for its	1. Principle of organ preservation, anatomical integrity, and unchanged organ function.
implementation	2. Principle of cavity consistency.
	3. Principle of aseptic technique.
	4. Principle of avoiding chemical irritant release.
	5. Principle of natural cavity channel preference.
	6. Principle of substitution under natural cavity channel contraindications.
	7. Principle of shortest surgical approach.
	8. Principle of bleeding prevention and control.
	9. Principle of perforation prevention and closure.
	10. Principle of tumor treatment.

SMIS, super minimally invasive surgery.

At different stages of DTTs, the SMIS treatment mode encompasses a spectrum of progressively advanced approaches. For intramucosal cancer with an extremely low risk of LNM, local non-FTR—achieved through endoscopic submucosal dissection (ESD) or endoscopic mucosal resection (EMR)—can provide radical removal with minimal invasion, followed by close surveillance. For lesions invading the submucosal layer, local progressive FTR may be performed via endoscopy or laparoscopy to achieve en bloc removal of the lesion and the full-thickness, which is suitable for selected high-risk early cancer(EC). In cases where LNM risk exists, local progressive FTR combined with precise lymph node.

## Methods

### Composition of the guideline development team

This clinical guideline was commissioned by the SMIS Committee of WEO and CSDE.

### Evidence review process and the formation of clinical recommendations

The working group conducted a structured systematic literature search based on keywords, covering nine literature databases: PubMed, Embase, Web of Science, Cochrane Central Register of Controlled Trials (CENTRAL), China National Knowledge Infrastructure (CNKI), China Science and Technology Journal Database, Wanfang Data, China Biomedical Literature Database, and Chinese Medical Database. The search included both Chinese and English articles published by January 1, 2025, and relevant monographs on minimally invasive surgery. The methodologists designed the search strategy. The studies included in this evidence-based guideline were arranged by evidence level in the following descending order: published systematic reviews/meta-analyses, randomized controlled trials (RCTs), prospective and retrospective observational studies, and case series. Bias risk and quality assessments followed the corresponding international evaluation standards: RCTs used RoB 2.0, non-randomized controlled intervention studies used ROBINS-I, and systematic reviews and meta-analyses used AMSTAR 2.^[6]^

The draft of the practice guidelines underwent multiple rounds of discussion and revision by the expert committee. The levels of recommendation and evidence were evaluated using the Grading of Recommendations, Assessment, Development, and Evaluations (GRADE) system (Table 3).^[7]^ The term “recommendation” is used to describe a “strong recommendation,” while “suggestion” or “consideration “is used to describe a “weak recommendation.” The consensus principle used for voting on the recommendations was as follows. (1) If the number of votes supporting a recommendation exceeded 50%, the direction (*e.g*., support or oppose the intervention) and strength of the recommendation were determined directly. (2) If this standard was not met but the total number of votes in favor of the same direction exceeded 70%, the direction of the recommendation was determined, and the strength of the recommendation was based on the highest number of votes. (3) If neither of these conditions was met, further discussions were conducted to reach an agreement. These guidelines were developed based on Appraisal of Guidelines for Research and Evaluation II (AGREE II)^[8]^ and Reporting Items for Practice Guidelines in Healthcare (RIGHT).^[9]^ Declarative viewpoints were expressed using a Good Practice Statement (GPS).

**Table 3:** GRADE, levels of evidence, and recommendations

Evidence Level	Specific Description	Study Types Total Score	Expression Symbol/Letter
High-level evidence	We are very confident that the true effect is close to the estimated effect.	• RCTs ≥ 0 • Observational studies with an increased quality of one level	⊕⊕⊕⊕/A
Moderate-level evidence	We have moderate confidence in the effect estimate. The true value may be close to the estimate, but there remains a possibility of a significant difference between the two.	• RCTs with a decreased quality –1 of one level • Observational studies with an increased quality of two levels	⊕⊕⊕○/B
Low-level evidence	We have limited confidence in the effect estimate. The true value may differ significantly from the estimate.	• RCTs with a decreased quality –2 of two levels • Observational studies	⊕⊕○○/C
Very low-level evidence	We have very little confidence in the effect estimate. The true value is likely to be quite different from the estimate.	• RCTs with a decreased quality ≤ –3 of three levels • Observational studies with a decreased quality of one level • Case series observations • Case reports	⊕○○○/D
**Recommendation Strength**		**Specific Description**	**Expression Symbol/Letter**
Strong recommendation to support the use of an intervention		The evaluator is confident that the benefits of the intervention outweigh the harms.	↑↑/1
Weak recommendation to support the use of an intervention		The advantages and disadvantages are uncertain, or the evidence, regardless of quality, shows that the advantages and disadvantages are roughly equal.	↑?/2
Weak recommendation to oppose the use of an intervention		The evaluator is uncertain about the advantages and disadvantages, or the evidence, regardless of quality, shows that the advantages and disadvantages are roughly equal.	↓?/2
Strong recommendation to oppose the use of an intervention		The evaluator is confident that the harms of the intervention outweigh the benefits.	↓↓/1

⊕⊕⊕⊕/A: Represents high-quality evidence. ⊕⊕⊕○/B: Represents moderate-quality evidence. ⊕⊕○○/C: Represents low-quality evidence. ⊕○○○/D: Represents very low-quality evidence. ↑↑/1: Indicates a strong recommendation for the use of a certain intervention. ↑? /2: Indicates a weak recommendation for the use of a certain intervention. ↓? /2: Indicates a weak recommendation against the use of a certain intervention. ↓↓/1: Indicates a strong recommendation against the use of a certain intervention. RCT, randomized controlled trials.

When evaluating the GRADE evidence quality, several factors may have led to either downgrading or upgrading the evidence quality level. Factors that downgraded evidence quality were limitations of the study (severe: -1 point, very severe: -2 points); inconsistency of study results (severe: -1 point, very severe: -2 points); uncertainty of direct evidence (partial: -1 point, complete: -2 points); inaccuracy or wide confidence intervals (severe: -1 point, very severe: -2 points); and the presence of publication bias (possible: -1 point, very likely: -2 points). Factors that may have upgraded the evidence quality level were effect size, which was large, +1 point (evidence from two or more studies consistently showing RR < 2 or RR < 0.5, with minimal confounding factors); or very large, +2 points (direct evidence showing RR < 5 or RR < 0.2, without affecting its validity). Additionally, the following were assigned based on factors that impacted effectiveness: confounding factors that may have reduced effectiveness, +1 point; and dose-effect relationship, +1 point (clear association between the dose of the drug and the effect size was present). These factors should be considered comprehensively when assessing evidence quality. Additional supporting materials, including the evidence summaries for each recommendation, are provided in the online Appendix (Supplementary Materials).

### Guide registration

These guidelines have been registered on the international practice guideline transparency platform (Practice Guideline Registration for Transparency, PREPARE) with the registration number PREPARE-2024CN1183.

## Results

**Section 1: Application, implementation rules, and nomenclature of SMIS in DTTs**

**Question 1**: Surgical models of SMIS for DTTs

**Recommendation 1**: In the treatment of DTTs, it is recommended that surgical procedures which preserve organs and physiological function without altering the body’s natural anatomical structure, and which are capable of achieving complete cure and restoring the preoperative state, be classified under SMIS.

(GPS; Recommendation level: strong recommendation; Level of agreement: 100%)

SMIS is defined as a surgical approach applied to diseases that require operative intervention or those with unsatisfactory outcomes from long-term pharmacological treatment. The fundamental principle is to remove or eradicate pathological lesions while preserving the integrity of the body’s natural anatomical structure, thereby achieving curative treatment.^[10]^ Throughout this development process, endoscopic diagnosis and treatment technologies have achieved breakthroughs in applications from within the digestive tract lumen to beyond the lumen, gradually expanding from the treatment of internal medicine diseases to surgical diseases, thus aligning more closely with the scope of surgical treatments. To better implement the concept of SMIS, deep multidisciplinary integration between the fields of digestive endoscopy and surgery is required, in which SMIS that extends beyond endoscopy is performed to ensure the maximum preservation of anatomical integrity while eradicating the disease.^[1,10,11]^

**Question 2**: Access routes for SMIS in DTTs

**Recommendation 2**: It is recommended that SMIS procedures be performed via four principal access routes—the natural cavity channel, the tunnel channel, the puncture channel, and the multi-cavity channel. These channels, in combination with diverse surgical techniques and technologies, enable precise and invasive therapeutic interventions.

(GPS; Recommendation level: strong recommendation; Level of agreement: 100%)

The direction of SMIS development is focused on surgical improvements related to the four types of channels, which include the continuous implementation and promotion of procedures with high safety and efficacy, efforts to improve procedures with high efficacy but low safety, and the continuous expansion of new SMIS techniques, methods, and therapeutic indications.^[4,12]^ The SMIS surgical techniques for DTTs are as follows:

1. SMIS *via* natural cavity channels. This refers to surgery performed through a naturally open passage of the human body that meets the super minimally invasive requirements. This includes non-FTR and FTR of the digestive tract, which are the preferred treatments for early DTTs. Active exploration and refinement of SMIS resection techniques for super indications and methods for closing large wound areas are under development. Procedures such as laser lithotripsy and stone removal while preserving the duodenal papilla, and endoscopic resection of precancerous lesions in pancreaticobiliary cancers under direct visualization, are also included in this category.

2. SMIS *via* tunnel channel. This refers to surgery performed through an artificially created tunnel in tissue, with the procedure taking place within the created space and meeting the super minimally invasive criteria. This includes mucosal-side nonFTR of extensive early DTTs, resection of tumors in the submucosal layer *via* a tunnel channel, and full-thickness incisions from the muscularis propria side of the digestive tract for treating conditions such as achalasia and gastroparesis, and tumor removal from the digestive tract lumen through the muscularis propria.

3. SMIS *via* puncture channel. This refers to surgery performed through a puncture channel into a cavity or lumen that meets the super minimally invasive criteria. Examples include endoscopic ultrasound (EUS)-guided percutaneous puncture, sclerotherapy for pancreatic cystic lesions, and percutaneous abdominal puncture FTR of DTTs, with or without LN dissection or removal.

4. SMIS *via* multi-cavity channel. This refers to surgery performed through more than one channel and/or using more than one endoscope within a cavity, in which access through all channels meets the super minimally invasive requirements. This approach may be used for accessing difficult-to-reach areas where a single endoscopic approach might fail, offering an alternative approach for challenging locations. For example, combined endoscopy and laparoscopy may be used to resect DTTs with or without LN dissection or removal.

**Question 3**: Principles for selecting the SMIS access route

**Recommendation 3**: To achieve the maximum benefit from super minimally invasive surgery, it is recommended that the procedure be conducted in adherence to the following principles: organ and function preservation, cavity consistency, aseptic technique, avoidance of chemical irritants, preference for natural cavity channels, substitution of natural cavity channels under contraindications, shortest surgical approach, prevention and control of bleeding, prevention and closure of perforations, and principles of tumor treatment.

(GPS; Recommendation level: strong recommendation; Level of agreement: 97%)

The implementation of SMIS follows a set of core principles aimed at ensuring the safety and efficacy of the surgical procedure through a comprehensive preoperative lesion assessment, precise selection of surgical techniques, and the prevention and management of complications. This approach seeks to provide patients with treatment outcomes and a quality of life consistent with the concept of SMIS.^[12,13]^ These core principles are as follows:

1. Principle of organ preservation, anatomical integrity, and unchanged organ function: The surgery focuses on removing the diseased tissue without damaging surrounding organs and tissues. Due to limitations, such as the depth and extent of the lesion, it may not be possible to remove only the diseased tissue. Efforts should be made to maximally preserve the surrounding organs and tissues while ensuring complete lesion excision, with anatomical structure and function maintained.

2. Principle of cavity consistency: The entry point, approach, and target location of the surgical procedure should ideally be within a single cavity, minimizing the need for multi-cavity incisions to reduce trauma and recovery time. In SMIS treatment, the use of four-channel access should be considered as the primary approach.

3. Principle of aseptic technique: Aseptic conditions should be the first priority, particularly in surgeries involving multi-cavity channels or percutaneous abdominal or thoracic puncture approaches. Strict adherence to aseptic principles is required.

4. Principle of avoiding chemical irritant release: In SMIS procedures involving punctures or multi-cavity channels, it is essential to avoid puncturing through ductal systems, such as blood vessels, lymphatic vessels, bile ducts, the gallbladder, and pancreatic ducts, to prevent the introduction of chemical irritants into the abdominal cavity, which could cause infection.

5. Principle of natural cavity channel preference: If there is an option to use a natural orifice, preference should be given to natural orifice routes, provided they comply with the first four principles.

6. Principle of substitution under natural cavity channel contraindications: When access through a natural orifice is contraindicated due to factors such as obstruction or narrowing, a puncture channel should be chosen, ensuring adherence to the first four principles.

7. Principle of shortest surgical approach: When selecting the surgical access route, consideration should be given to the distance between the entry point and the surgical site. The shortest possible path should be chosen to minimize damage to surrounding organs or tissues, reduce surgical difficulty, and shorten the procedure time.

8. Principle of bleeding prevention and control: Preoperative assessment of bleeding risk should be thorough, and effective preventative and hemostatic contingency measures should be used to ensure an almost 100% hemostasis success rate. This principle is essential in the application of the four-channel access in SMIS procedures.

9. Principle of perforation prevention and closure: Prior to surgery, perforation risk should be thoroughly assessed, and appropriate techniques for prevention and closure should be applied to restore the integrity and closure of the body’s natural cavities. This principle must be considered in the application of the four-channel access in SMIS procedures.

10. Principle of tumor treatment: For SMIS treatment of benign and malignant tumors, principles such as not cutting through the tumor, en bloc resection, tumor-free techniques, and prevention of metastasis should be followed.

**Question 4**: Nomenclature for SMIS in DTTs

**Recommendation 4**: The nomenclature convention for SMIS should include the lesion location, lesion nature, surgical channel, and treatment method, such as “lesion location+lesion nature+channel+super minimally invasive resection.”

(GPS; Recommendation level: strong recommendation; Level of agreement: 96%)

To succinctly and comprehensively summarize the disease and its treatment method in a manner reflecting its application that is easily recognized, the currently established SMIS nomenclature convention is as follows. The nomenclature must include the lesion location, lesion nature, surgical channel, and treatment method.^[5]^ The following nomenclature convention is recommended for adoption: “lesion location + lesion nature + channel + super minimally invasive excision/incision/removal/drainage/ ablation”. This convention represents the final diagnosis after the surgery is completed and pathological results are integrated. Previously used endoscopic techniques, such as endoscopic mucosal resection (EMR) and endoscopic submucosal dissection (ESD), which are SMIS methods, are not included in the surgical nomenclature but should be described in detail in the surgical record, including the treatment process and specific techniques applied. For example, the natural cavity channel (per-oral SMIS-FTR for GC on the greater curvature of gastric body), the tunnel channel (per-tunnel SMIS-non FTR for GC on the greater curvature of gastric antrum), the puncture channel (per-puncture SMIS-FTR for appendiceal adenoma), and the multi-cavity channel (per-multi-cavity SMIS for GC on the anterior wall of gastric body).

**Section 2: The application of SMIS in treating early esophageal cancer (EEC) and precancerous lesions, along with strategies for stricture prevention**

**Question 5**: For patients with esophageal dysplasia or early stage, well-differentiated, non-ulcerative EEC, it is recommended to select super minimally invasive non-full-thickness resection (nft-SMIR).

**Recommendation 5**: For patients with esophageal dysplasia or early, well-differentiated, non-ulcerative EC, it is recommended to select non-full-thickness SMIS resection based on the lesion size and circumferential extent. When the lesion involves more than half of the esophageal circumference, endoscopic submucosal tunnel dissection (ESTD) is advised.

(Evidence level: High; Recommendation level: strong recommendation; Level of agreement: 87%)

In the treatment of EEC and precancerous lesions, SMIS provides a treatment option that preserves organ integrity. Key factors influencing the selection of the resection method include the tumor size, depth of invasion, histological grading, and presence of ulcers. The American Society for Gastrointestinal Endoscopy (ASGE) guidelines for ESD in the treatment of early esophageal and GC recommend a lesion size of 15 mm as the cutoff to decide between ESD and EMR. However, this should not be seen as a strict threshold, as there is no upper limit established for lesion size with ESD.^[14]^ Additionally, determining the depth of invasion before resection is essential. The Japanese Esophageal Society’s guidelines for EC treatment define absolute indications for ESD as flat lesions (Paris 0–II), invasion into M1 to M2, and no more than two-thirds circumferential involvement, while relative indications are M3 to SM1 lesions, in which endoscopic resection would leave a more than 75% circumferential mucosal defect.^[15]^ Ensuring the resection of an entire specimen is crucial for assessing cure rates, accurate staging, guiding further treatment decisions, and managing recurrence after resection.

ESTD has been shown to be highly effective and safe for treating large early-stage esophageal cancers. Compared to traditional ESD, ESTD has a higher complete resection rate, faster dissection speed, and lower postoperative recurrence rate. Therefore, ESTD can be considered the first choice of SMIS for large early-stage EC. The decision to use SMIS resection should also be based on several other factors, including local expertise, availability, the judgment of the endoscopist, and patient preferences.

**Question 6**: Postoperative stricture prevention in esophageal SMIS

**Recommendation 6**: For patients with circumferential esophageal defects ≥75%, it is recommended to implement systematic perioperative interventions for stricture prevention and to establish a stepwise management protocol based on endoscopic monitoring, in order to achieve precise prevention and control of esophageal stricture.

(Evidence level: Medium; Recommendation level: strong recommendation; Level of agreement: 94%)

Currently, methods for preventing esophageal stricture include the following: (1) Prophylactic balloon dilation (EBD): EBD can reduce the incidence of post-ESD esophageal stricture and shorten the treatment time required after stricture occurs. However, EBD requires repeated endoscopic procedures, causing significant discomfort and an economic burden for patients, and is therefore not recommended for standalone use. (2) Esophageal stents: Esophageal stents can be used as a preventive measure, but care must be taken to avoid potential complications such as stent displacement and restenosis. (3) Corticosteroids: The local use of corticosteroids can suppress inflammatory responses and the synthesis of collagen fibers and promote collagen breakdown, thereby reducing the incidence of post-ESD stricture. (4) Scar inhibitors and wound coverage: The use of scar inhibitors and wound coverage techniques can reduce scar formation and prevent stricture.

(5) Autologous mucosal grafting: Autologous mucosal grafting is an innovative method that has been used to prevent stricture after endoscopic treatment of esophageal circumferential lesions with good results.

**Section 3: Surgical selection and curative assessment of SMIS for EGC and precancerous lesions**

**Question 7**: For patients with EGC or gastric epithelial dysplasia or HGIN, SMIS can be performed.

**Recommendation 7**: For patients with EGC or gastric epithelial dysplasia/high-grade intraepithelial neoplasia, SMIS is recommended when the evaluation of LN status can be accurately controlled.

(Evidence level: Moderate; Recommendation level: strong recommendation; Level of agreement: 90%)

With the increasing detection rate of EGC, its proportion in the overall cases of GC has approached 20%.^[16]^ Radical gastrectomy combined with systematic lymph node dissection remains the preferred surgical approach in some regional hospitals. However, the regional LN status in the surgical scope is a key factor and crucial component in determining both the surgical approach and prognosis.^[17]^ Previous studies have shown that this type of surgery can effectively control cancer recurrence; however, many patients without LNM unnecessarily suffer from complications and sequelae caused by organ resection. Moreover, the extent of necessary LN dissection remains a subject of ongoing debate.^[18, 19, 20]^ The removal of non-metastatic LNs may disrupt the immune system, potentially causing secondary effects on the primary tumor or latent metastatic foci.^[17, 18, 19, 20, 21]^ Given that excessive organ and LN resection can cause irreversible harm to the body, it is of great significance to choose and perform less invasive, more individualized, and precise surgical strategies.^[22]^

In recent years, the concept of SMIS has significantly transformed the treatment approach and surgical paradigms of EGC to become the first-line treatment option for both EGC and precancerous lesions. The surgical techniques include the following: (1) nft-SMIR, which includes treatments that have been widely developed and used for decades, such as ESD, ESTD, EMR, and endoscopic intermuscular dissection (EID), with the dissection layer extending from the mucosal layer to the muscularis propria, and the recently developed stepwise super minimally invasive non-full-thickness resection (snft-SMIR); (2) super minimally invasive full-thickness resection (ft-SMIR) using endoscopy, including stepwise super minimally invasive full-thickness resection (sft-SMIR), endoscopic full-thickness resection(EFTR), and hybrid EFTR; and (3) super minimally invasive multi-cavity channel gastrectomy, which involves performing full-thickness lesion resection combined with LN dissection using an endoscope *via* natural cavity channels and a laparoscope through a percutaneous abdominal puncture channel. These three surgical methods must be chosen based on the strict T-stage classification of gastric cancer, ensuring maximal patient safety and quality of life while maintaining curative intent.^[22]^

A preliminary exploration of the LN dissection range of EGC in different tumor locations for super minimally invasive multi-cavity channel gastric conserving surgery, and the clinical practice experience obtained from the original clinical guidelines are summarized as follows.

1. Lower middle GC (distal GC) : (1) The original guidelines recommend D1 dissection, *i.e*., No. 1 (right cardia), 3 (lesser curvature side), 4sb (left gastric omentum vascular side), 4 d (right gastric omentum vascular side), 5 (upper pylorus), and 6 (lower pylorus). (2) Local FTR with regional LN dissection was classified according to specific location (ultimately based on the actual location of the tumor) as follows: ① antrum of the stomach, No. 3 (lesser curvature side), 4sb (adjacent to the left vessel of the gastric omentum), 4 d (adjacent to the right vessel of the gastric omentum), 5 (above the pylorus), 6 (below the pylorus), and 7 (adjacent to the left gastric artery); ② small curvature side of gastric body, No. 1 (right cardia), 3 (small curvature side), 5 (upper pylorus), and 7 (adjacent to left gastric artery); and ③ on the greater curvature side of the gastric body, No. 4sb (adjacent to the left vessel of the gastric omentum) and 4 d (adjacent to the right vessel of the gastric omentum).

2. Upper GC (proximal GC): (1) The original guidelines recommend D1 dissection, *i.e*., No. 1 (right cardia), 2 (left cardia), 3a (upper part of lesser curvature), 4sa (adjacent to short gastric vessels), 4sb (adjacent to left gastric omentum vessels), and 7 (adjacent to left gastric artery). (2) Local FTR with regional LN dissection was classified according to specific locations (ultimately based on the actual location of the tumor) as follows: ① lesser curvature side of the gastric fundus, No. 1 (right of the cardia), 2 (left of the cardia), 3 (lesser curvature side), and 7 (adjacent to the left gastric artery); and ② gastric fundus vault, No. 1 (right cardia), 2 (left cardia), and 4sa (near the short blood vessels of the greater curvature of the stomach).

**Question 8**: For patients with EGC undergoing SMIS, a precise assessment of LNM risk should be performed, and targeted LN dissection may be conducted when feasible.

**Recommendation 8**: For patients with EGC or high-grade gastric intraepithelial neoplasia, it is recommended that the SMIS resection strategy be selected based on the tumor’s characteristics and stratified LNM risk. In addition, comprehensive decision-making should take into account the patient’s life expectancy, physical condition, social factors, and personal preferences.

(Evidence level: Moderate; Recommendation level: strong recommendation; Level of agreement: 94%)

Due to the complex and multidirectional lymphatic drainage pattern of the stomach, SLN detection, which targets the first station (D1) of the primary tumor’s lymphatic drainage pathway, can yield false negatives. This presents a clinical challenge in digestive tract LN tracing. However, researchers have proposed various methods to predict LNM in multiple studies. Currently, non-invasive preoperative detection methods, including PET-CT and CT of the chest, abdomen, and pelvis combined with ultrasound of superficial human LNs, are effective approaches for assessing the systemic status of the tumor and LNM.^[23]^ Patients with EGC carry a risk of LNM, though reported rates vary across centers. The incidence of LNM in intramucosal cancer is relatively low, ranging from approximately 2% to 5.9%, while the rate increases in submucosal cancer (T1b stage), ranging from about 2.5% to 20.3%.^[24, 25, 26, 27]^ The submucosal layer is rich in lymphatic vessels, and once cancer infiltrates this layer, the likelihood of LNM *via* lymphatic reflux rises significantly. Therefore, greater attention is given to the LNM rate in T1b EGC. For example, Abdelfatah ^[26]^ reported an LNM rate of only 2.5% in T1b cases, whereas the General Surgery Department of the PLA General Hospital reported a rate as high as 20.3%.^[27]^ In its second edition of the endoscopic treatment guidelines for EGC, the Japan Gastroenterological Endoscopy Society (JGES)^[28]^ proposed a stratified evaluation of LNM rates based on tumor size, histological type, and the presence or absence of ulceration, which estimated the risk to range from 1% to 10.6% (Table 4).

**Table 4:** Risk of lymph node metastasis after radical resection of EGC with ESD

	T1a				T1b	
Tumor feature	No ulcer		With ulcer		Submucosal infiltration	
					< 500 μm	≥ 500 μm
Size	≤ 2 cm	< 2 cm	≤ 3 cm	< 3 cm	≤ 3 cm	No size limitation
Differentiated type	< 1%	< 1%	< 1%	3.0%	2.6%	1%–9%
Undifferentiated type	< 1%	2.8%	5.1%		10.6%	

EGC, early gastric cancer; ESD, endoscopic submucosal dissection.

**Figure 1:**
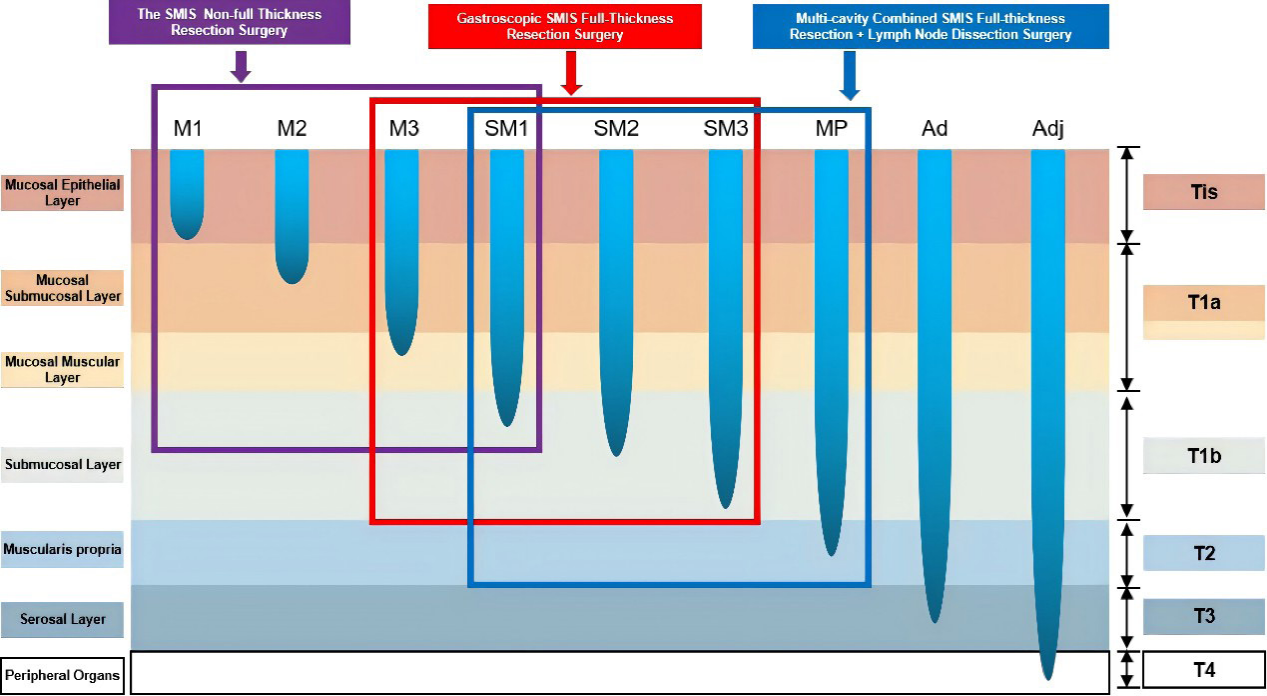
SMIS surgical selection strategy for early gastric cancer. SMIS, super minimally invasive surgery.

For Tis, T1a-M1, and T1a-M2 EGC, non-full-layer resection or full-layer resection is used. For some T1a-M3 and T1b EGC, full-thickness resection surgery or multi-cavity SMIS gastric preservation surgery is considered. For T1b with high risk factors of LNM or T2 with a low risk of LNM, multi-cavity SMIS gastric conserving surgery should be considered (Figure 1).

**Question 9**: Evaluation of curative effect for SMIS in EGC

**Recommendation 9**: For patients with EGC or high-grade gastric intraepithelial neoplasia, it is recommended to apply the SMIS-cure evaluation system to perform a stratified assessment of curative outcomes, and to develop individualized adjuvant treatment plans and prognostic management strategies based on the evaluation results.

(Evidence level: Low; Recommendation level: strong recommendation; Level of agreement: 97%)

The evaluation of post-EGC cure has significant clinical value, as evidenced by the following: (1) guiding subsequent treatment, where the correct selection of chemotherapy, radiotherapy, or targeted therapy reduces the risk of recurrence; (2) assessing recurrence risk, where formulation of more targeted follow-up plans and monitoring strategies; (3) improving patient quality of life, in that for patients with a higher cure rate, unnecessary subsequent treatments can be reduced, thus minimizing treatment-related side effects; (4) psychological support, in that a cure evaluation provides patients with a clear treatment outcome, thus helping alleviate anxiety and uncertainty and boosting their confidence in the future; (5) health economic considerations, where a cure evaluation helps patients and doctors assess the economic benefits of treatment, avoiding unnecessary treatment costs and alleviating the patient’s financial burden; (6) prognostic communication, that is, a cure evaluation offers a quantifiable prognostic indicator, facilitating more effective communication between doctors, patients, and their families, thus helping them gain a clearer understanding of the treatment outcome; (7) health decision-making, in that patients can make more informed health decisions based on the cure evaluation results, such as whether to modify their lifestyle or dietary habits to reduce the risk of recurrence; (8) research participation, in that patients with unsatisfactory cure evaluation results may be more motivated to participate in clinical studies to seek new treatment methods; and (9) legal and insurance matters, where, in some cases, the cure evaluation result may affect the patient’s legal rights and insurance claims.

With the expansion of treatment indications for EGC and the iterative innovations in related SMIS surgical techniques, the limitations of the traditional eCura evaluation system in clinical applications have become increasingly apparent. To address this, Professor Enqiang Linghu’s team introduced the SMIS-Cure evaluation system in 2025 (Table 5). By integrating key factors, such as surgical method selection, tumor size, depth of invasion, differentiation type, ulceration status, vascular and nerve invasion, and residual lesions in the surgical specimen, the system forms a three-level dynamic evaluation standard: SMIS-Cure A (cured), SMIS-Cure B (clinically cured), and SMIS-Cure C (surgical reassessment). The evaluation also includes LNM risk assessment prior to the preoperative evaluation to create a more systematic evaluation framework that aligns with the characteristics of SMIS surgery.

**Table 5:** Early gastric cancer SMIS-Cure evaluation system (version 1)

SMIS-Cure A (cured)	Specific tumor characteristics are met with no evidence of lymph node metastasis (LNM) in the preoperative assessment after SMIS non-full-thickness or full-thickness resection is performed. The postoperative pathological examination confirms no residual lesions at the base and margin of the specimen, and no vascular or neural invasion. EGC is then classified as SMIS cured, with routine postoperative follow-up.
	Tumor characteristics:
	(1) Differentiated intramucosal cancer without ulceration, regardless of the tumor diameter.
	(2) Undifferentiated intramucosal cancer without ulceration, with a tumor diameter ≤ 2 cm.
	(3) Differentiated submucosal cancer with or without ulceration, with a tumor diameter ≤ 3 cm: SM1, with an infiltration depth < 500 μm.
SMIS-Cure B (clinically cured)	This is divided into two subgroups, B1 and B2. Both subgroups need to meet specific tumor characteristics, with no evidence of LNM before surgery. After undergoing SMIS full-thickness resection or SMIS stomach-preserving resection through a multi-cavity channel (full-thickness resection of the local lesion with regional lymph node resection), if the postoperative pathological examination confirms no residual lesion at the base and surgical margins of the specimen, no vascular or nerve invasion, and no LNM is found, the EGC can be determined to be SMIS clinical cure. Intensive and close follow-up is required after the operation.
	B1: The tumor characteristics are as follows:(1) Differentiated submucosal cancer with a tumor diameter ≤ 3 cm: SM2, with an infiltration depth of 500–1000 μm. (2) Undifferentiated submucosal cancer with a tumor diameter ≤ 2 cm: SM1, with an infiltration depth < 500 μm. In such cases, the EGC is classified as SMIS-Cure B1.
	B2: The tumor characteristics are as follows: (1) Differentiated submucosal cancer with a tumor diameter ≤ 3 cm: SM3, with an infiltration depth < 1000 μm. (2) Undifferentiated submucosal cancer with a tumor diameter ≤ 2 cm: SM2, with an infiltration depth of 500–1000 μm. Chemotherapy and/or immunotherapy should be supplemented after the operation, and after completing two rounds of intensive and close follow-up, the ECG can be classified as SMIS-Cure B2.
SMIS-Cure C (surgical reassessment)	For those with suspected postoperative residue or high-risk factors (vascular and nerve invasion or discovery of LNM after the operation), re-evaluation of surgery is required. This includes three subgroups, C1, C2, and C3.
	C1: Residual lesion is present at the base and/or surgical margin of the SMIS resection specimen, there is no vascular or nerve invasion, and no LNM is found. Reexamination by endoscopy is required, and multiple biopsies should be obtained at the residual end of the wound edge and/or the basal surface. If the pathological result is negative, no residual lesion at the wound surface is determined, and close endoscopic follow-up is required.
	C2: Residual lesion is present at the base and surgical margin of the SMIS resection specimen, there is no vascular or nerve invasion, and no LNM is found. If the pathological result of the standardized biopsy of the wound surface is positive, additional surgery must be performed, and intensive and close follow-up is required after the operation. The SMIS surgical method is selected according to the specific tumor characteristics.
	(1) If the tumor conforms to the characteristics of SMIS-Cure A and there is only marginal residue in the postoperative specimen, SMIS partial-thickness or full-thickness resection should be supplemented.
	(2) If the tumor conforms to the characteristics of SMIS-Cure B and there is only marginal residue in the postoperative specimen, SMIS full-thickness resection should be supplemented.
	(3) If the tumor conforms to the characteristics of SMIS-Cure A (3) and there is a tumor residue at the base of the submucosa in the postoperative specimen, SMIS stomach-preserving resection through a multi-cavity channel should be supplemented.
	(4) Regardless of the tumor characteristics, if there is tumor residue at the base of the muscularis propria in the postoperative specimen, standard radical surgical resection should be supplemented.
	C3: The pathological result of the surgical specimen indicates vascular and nerve invasion, or LNM is found after stomach-preserving resection through a multi-cavity channel, or it does not conform to SMIS-Cure A/B/C1/C2. Standard radical surgical resection must be performed in the later stage.

Notes. 1. Definition of EGC: The cancerous tissue is confined to the gastric mucosa layer or submucosa layer, regardless of the presence or absence of LNM. 2. Intensive and close follow-up: Follow-up at 3-month intervals using a combination of imaging and endoscopy is required. 3. Close follow-up: Endoscopic follow-up at 3-month intervals is required. This is the first edition of the evaluation system. With the clinical popularization of SMIS and the continuous accumulation of evidence-based medical data on EGC, the SMIS-Cure evaluation system will be dynamically updated in combination with technological progress and clinical practice needs to ensure that it keeps pace with medical developments and increasingly provides a more accurate diagnosis and treatment basis for patients. SMIS, super minimally invasive surgery; EGC, early gastric cancer; LNM, lymph node metastasis.

Within the preoperative stratification assessment, the LNM risk is divided into three levels based on multimodal imaging diagnostic results. (1) No evidence of LNM metastasis: no tumor-related LNs are found using PET-CT, abdominal enhanced CT, or magnetic resonance imaging (MRI) combined with whole-body superficial LN ultrasound. (2) Suspicious LNM risk: abdominal CT, MRI, or whole-body superficial lymph node ultrasound suggests the presence of lymph nodes but cannot confirm whether they are tumor-related, requiring combined PET-CT examination, which still cannot completely rule out the possibility of tumor-related LN. (3) Clear evidence of LNM: through one or more imaging modalities, such as abdominal CT, MRI, whole-body superficial LN ultrasound, or PET-CT, the presence of tumor-related LNs is confirmed. Compared with the eCura grading system, the innovative breakthrough of SMIS-Cure lies in the introduction of new surgical evaluation standards, such as ft-SMIR and SMIS multi-cavity gastric preservation surgery, which cover the entire process from preoperative assessment to postoperative follow-up in an evidence-based decision-making framework, and retain the eCura A evaluation standard of the eCura system as the radical cure standard. The SMIS-Cure evaluation system aims to shift GC diagnosis and treatment from a “radical priority” model to a “radical and functional balance” paradigm.

**Question 10**: Postoperative follow-up strategy for SMIS in EGC

**Recommendation 10**: For patients with EGC or high-grade gastric intraepithelial neoplasia, the SMIS-cure evaluation system should be applied for stratified assessment, upon which individualized adjuvant treatment and follow-up management strategies should be formulated.

(Evidence level: Medium; Recommendation level: strong recommendation; Level of agreement: 96%).

**Figure 2:**
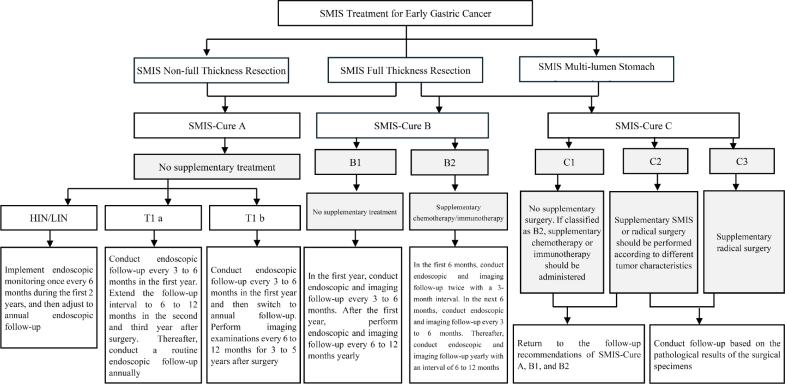
Supplementary treatment status after surgery evaluated by the SMIS-Cure system. SMIS, super minimally invasive surgery.

For patients with EGC and HGIN of the stomach, it is recommended that the characteristics of the three levels of cure degrees [SMIS-Cure A (cured), B (clinically cured), and C (surgical reassessment)] are systematically evaluated using the SMIS-Cure evaluation system, and additional individualized treatment and follow-up plans are formulated based on the grading results (Figure 2). Specifically, patients assessed as SMIS-Cure A can undergo standardized follow-up (endoscopic monitoring at intervals of 6–12 months), those at the SMIS-Cure B level require intensified and close follow-up (imaging combined with endoscopic evaluation every 3 months), and those at the SMIS-Cure C level require a multidisciplinary consultation to determine a secondary intervention strategy, while dynamically monitoring the risk of LNM and the effect of organ function preservation.^[119,120]^ For special populations who cannot tolerate surgery due to advanced age or severe complications, clinically, it is necessary to focus on evaluating the risk of LNM and the possibility of local recurrence and distant metastasis. It is essential to fully inform the patient that recurrent GC is usually incurable and has an extremely poor prognosis. This stratified decision-making process needs to take into account the individual situation of each patient to strike a balance between risk control and quality of life, and also give consideration to the rational utilization of medical resources.

**Section 4: SMIS treatment for early colorectal cancer (ECRC) and precancerous lesions**

**Question 11**: For patients with ECRC or high-grade colorectal intraepithelial neoplasia, SMIS can be performed.

**Recommendation 11**: For patients with ECRC or high-grade colorectal intraepithelial neoplasia without LNM, SMIS is recommended as an appropriate treatment option.

(Evidence level: High; Recommendation level: strong recommendation; Level of agreement: 99%).

In the past 15 years, there has been a tremendous transformation in the treatment model of colorectal tumors, with a gradual shift from traditional surgery to minimally invasive endoscopic techniques. With the application of new technologies and concepts, such as preoperative precise diagnoses, adjuvant chemoradiotherapy, immunotherapy, transanal endoscopic microsurgery (TEM), transanal endoscopic operations (TEO), transanal minimally invasive surgery (TAMIS), EFTR, and EID, the treatment model of CRC has gradually shifted towards preserving organ function.^[29]^ In recent years, a new treatment strategy concept referred to as “resection and analysis” has emerged for CRC stages T1 and T2. The resection and analysis strategy requires endoscopic resection, followed by pathological evaluation to assess the risk of LNM and the necessity of additional intestinal resection. Its use has provided further evidence of the oncological and endoscopic technical safety requirements for practical implementation.^[30,31]^

In recent years, surgical projects based on the concept of SMIS have included the following. (1) nft-SMIR surgery: treatments such as ESD, ESTD, EMR, and EID, which have been widely performed and developed for decades, have expanded the peeling layer from the mucosal layer to the muscularis propria. Furthermore, a new SMIS procedure, nsft-SMIR, enables the implementation of a progressive resection strategy that conforms to the growth characteristics of tumors. (2) ft-SMIR of digestive endoscopic lesions: a new SMIS procedure, the sft-SMIR, and the SMIS closure method of cutting and closing simultaneously provide a means of safe and effective FTR surgery performed through a single colonoscope *via* a natural body cavity. In addition, transanal local excision (LE) surgeries such as TEM, TEO, and TAMIS also conform to the concept of SMIS. (3) ft-SMIR of lesions combined with LN resection surgery: SMIS is carried out by combining the natural body cavity channel of the digestive endoscope with the percutaneous abdominal puncture channel of the laparoscope. These three surgical methods must be selected based on the strict T staging of CRC to ensure the patient’s safety and quality of life to the greatest extent.^[32, 33, 34, 35, 36]^

**Question 12**: For patients with CRC, preoperative assessment of LNM is an essential prerequisite for performing SMIS.

**Recommendation 12**: It is recommended that the SMIS resection strategy be selected based on the tumor’s characteristics and stratified LNM risk, while also making comprehensive decisions that consider the patient’s life expectancy, physical condition, social factors, and personal preferences.

(Evidence grade: High; Recommendation level: strong recommendation; Level of agreement: 99%).

Approximately 30% of patients initially present with CRC at Tis and T1 stages without LN involvement or metastasis, and surgery remains the standard treatment method for these patients. Currently, non-invasive preoperative detection methods, including PET-CT and enhanced CT of the chest, abdomen and pelvis combined with ultrasound examination of superficial LNs of the human body, are effective means of assessing the systemic status of tumors and LNM.^[23,35]^ In addition, rectal EUS and enhanced MRI are accurate tools for preoperative staging of rectal cancer (RC).^[37,38]^ Approximately 10% of T1 colorectal patients have LNM, among which the proportion of patients with pedunculated tumors (3–7%) is lower than that of those with sessile tumors (up to 28%).^[39,40]^ The risk of LNM can be stratified according to various histopathological factors, such as the depth of invasion, lymphovascular invasion, histological grade, and tumor budding.^[41,42]^

T1 CRC patients with a low risk of LNM can be cured by LE through SMIS, while high-risk T1 CRC requires radical surgery with lymph node dissection.^[43]^ Currently, many patients still undergo unnecessary radical surgery. Although most patients achieve radical cure, their quality of life in the later stage is sacrificed. New diagnostic techniques for LNM have been recently developed, such as nomograms, artificial intelligence systems, and genomic analysis, which can identify more low-risk T1 CRC cases.^[44,45]^ In addition, studies based on LE show that the curability and necessity of additional treatments (including postoperative chemoradiotherapy and preoperative neoadjuvant therapy) are becoming an acceptable strategy for T1 CRC, particularly for RC.^[46]^

When formulating the SMIS treatment plan, in addition to weighing the advantages and disadvantages of the treatment strategy based on the LNM risk stratification, the best SMIS surgical method should also be selected according to the LNM risk, as well as the patient’s life expectancy, physical condition, social characteristics, and personal wishes. According to the data from a multi-center study in the United States, the postoperative mortality rate of patients who received radical resection of rectal cancer increased significantly with age: it was only 1.5% for those aged 69 and below, but climbed to 8.0% for patients over 85 years old. A cross-national data comparison further showed regional differences in the postoperative mortality rate of surgery, with 1.3% reported in a Japanese cohort and 2.4% in a Dutch cohort. Therefore, for patients with a risk percentage equal to or lower than these reported risks, the risk of LNM is acceptable, and it is feasible to perform SMIS local resection surgery.

**Question 13**: Evaluation of curative outcomes and follow-up strategy for SMIS in CRC or high-grade intraepithelial neoplasia

**Recommendation 13**: For patients with CRC or high-grade intraepithelial neoplasia who undergo direct SMIS resection, the curative outcome should be evaluated based on whether en bloc R0 resection has been achieved in the pathological specimen. This serves as the core criterion for determining adjuvant therapy planning and prognostic assessment. Postoperative follow-up is generally recommended at 6 months after the procedure.

(Evidence level: High; Recommendation level: strong recommendation; Level of agreement: 97%)

The cure rate of early colorectal cancer is relatively high, usually ranging from 60 to 80%. Some studies even indicate that the cure rate of early colorectal cancer (particularly stage I or II) after surgical resection can exceed 90%. For extremely early intramucosal cancer, an almost 100% cure rate can be achieved.^[47]^

**Figure 3:**
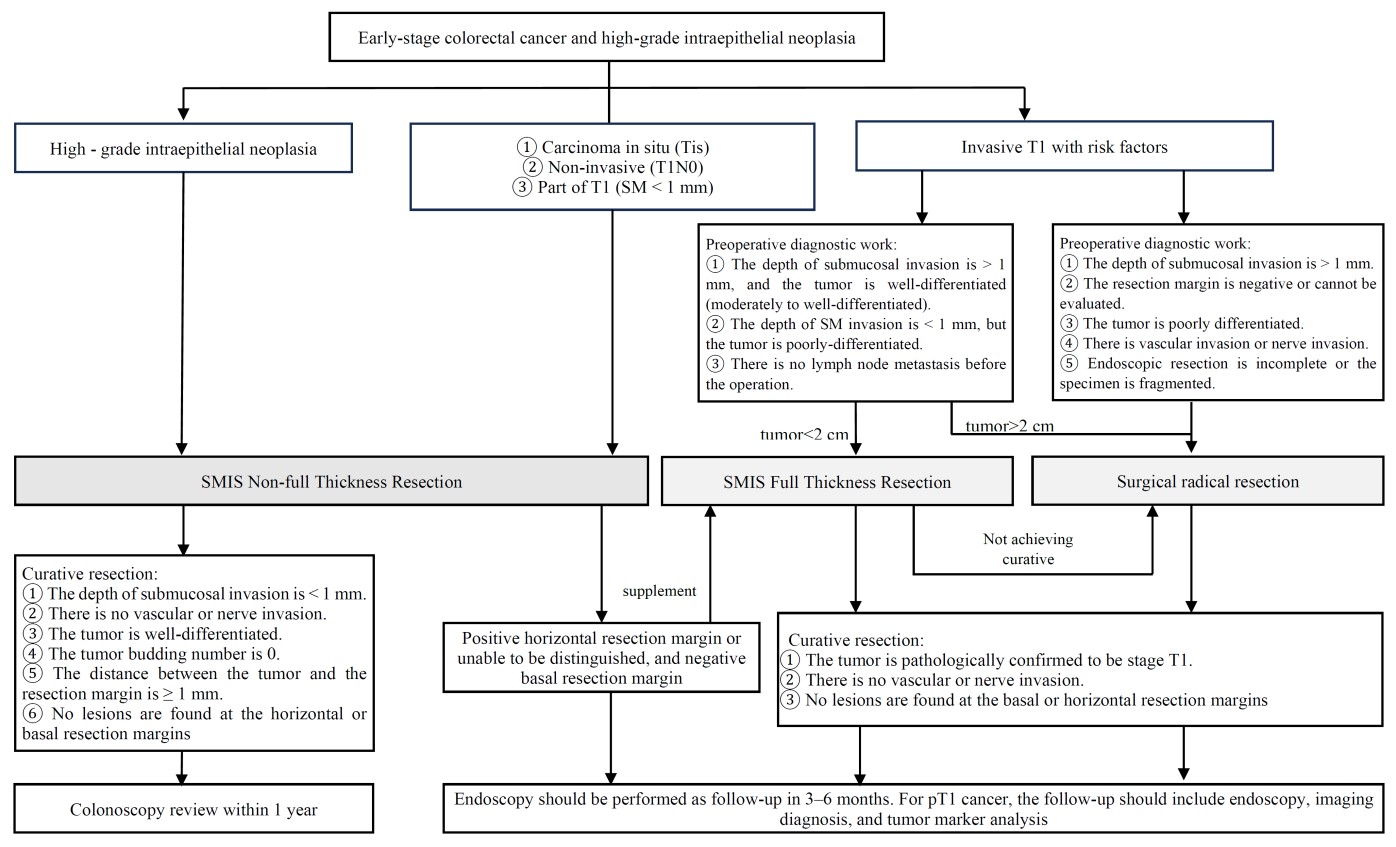
SMIS strategy selection, prognosis, and follow-up for early colorectal cancer and high-grade intraepithelial neoplasia. SMIS, super minimally invasive surgery.

The purpose of follow-up after SMIS for ECRC is to detect local residual tumor, recurrence, metastasis, and ectopic lesions at an early stage. The formulation of the follow-up plan should comprehensively consider various factors, such as the SMIS treatment techniques employed (such as en bloc resection or piecemeal resection), the assessment of resection integrity based on the pathological results of the specimens, the presence of recurrence risk factors, and the patient’s underlying diseases ^[48]^ (Figure 3). Currently, there is a lack of high-quality evidence-based medicine regarding the optimal follow-up interval after endoscopic resection of early CRC. According to the expert consensus, it is recommended that colonoscopy be performed 3, 6, and 12 months after curative endoscopic resection of early CRC. Imaging examinations (chest and abdominal CT) should also be performed to rule out the possibility of LNM and other conditions. If all reexamination results are normal, the follow-up interval can be extended to 1 or 3 years, with attention paid to combining tumor marker testing, fecal occult blood testing, and relevant imaging examinations. For lesions resected through piecemeal resection, it is advisable to conduct the first reexamination within 3 to 6 months according to different recurrence risks, and care should be taken to avoid a missed diagnosis of the lesion during follow-up. More detailed and effective follow-up plans require the support of more high-quality clinical studies in the future.^[49,50]^

**Question 14**: For selected patients with LARC who achieve tumor regression after neoadjuvant therapy, SMIS local excision may be considered.

**Recommendation 14**: For patients with locally advanced low RC who achieve a clinical complete response (cCR) or near-complete response (nCR) following neoadjuvant chemoradiotherapy with or without immunotherapy, it is recommended that, after thorough evaluation and confirmation of oncologic efficacy, an SMIS FTR be performed to assess the pathological response and guide subsequent treatment decisions

(Evidence level: Medium; Recommendation level: strong recommendation; Level of agreement: 70%).

The key points in the diagnosis of LARC include endoscopic or imaging findings of the primary rectal tumor, pathological confirmation of cancer, a tumor location within 12 cm of the anus, invasion beyond the muscular layer of the intestinal wall into the surrounding tissues or LNM, and the absence of distant metastasis.^[51]^ Low RC accounts for the majority of RC patients in China, and in its treatment, there are challenges in “saving lives, preserving the anus, and maintaining functions”.^[52]^ Currently, the standard treatment for LARC is the “sandwich” model, neoadjuvant chemoradiotherapy (nCRT) with total mesorectal excision (TME) and postoperative adjuvant chemotherapy.^[52,53]^ The Chinese diagnosis and treatment specifications ^[52]^ recommend neoadjuvant chemoradiotherapy for patients with stage II and III middle and low RC, including long-course chemoradiotherapy or short-course radiotherapy combined with chemotherapy, with chemotherapy regimens such as capecitabine being preferred.

TME is the gold standard for RC surgery. However, the surgery is technically difficult, may cause complications, and may also affect sexual function.^[52,53]^ For patients who achieve clinical complete response (cCR) or ncCR after neoadjuvant chemoradiotherapy, watch-and-wait (W& W) is a potential strategy and a current research direction. However, its local recurrence rate is relatively high, and the disease-free survival rate, overall survival rate, and disease-specific survival rate are not as high as those following total mesorectal excision. Although some studies have shown that a strict W& W strategy can detect local recurrence at an early stage without increasing the risk of systemic disease or resulting in poor survival outcomes,^[54]^ during the monitoring process, the uncertainty of clinical results can place a sizeable psychological and economic burden on patients and their families. From a pathological perspective, the W& W strategy does not completely eradicate the tumor tissue, and the remaining scar tissue provides conditions for local tumor recurrence.

LE, which involves resecting the tumor scar through transanal endoscopy, preserves the rectum, reduces the risk of local recurrence, and is helpful in determining a pathological complete response.^[55]^ SMIS with FTR may become a strong competitor to replace the standard of TEM in the treatment of LARC.^[56]^ However, while this technique resects the lesion, it can be accompanied by active perforations and defects in the intestinal wall. The closure of rectal wall defects remains a controversial issue. Clinical studies comparing the open and closed management of intestinal wall defects after local FTR of rectal tumors have found that there are no significant differences between the unclosed and closed groups in terms of the overall incidence rate, postoperative local infection rate, postoperative bleeding rate, and postoperative reintervention rate.^[57]^

**Section 5: SMIS treatment for duodenal papillary adenoma**

**Question 15**: For patients with benign or high-grade neoplastic duodenal papillary tumors that do not involve the bile duct or pancreatic duct, SMIS can be performed.

**Recommendation 15**: For patients with benign or high-grade neoplastic duodenal papillary tumors that do not invade the bile duct or pancreatic duct, it is recommended to perform per-oral SMIS resection of the duodenal papillary lesion as the initial treatment. Postoperative follow-up strategies and the decision on whether to proceed with additional pancreaticoduodenectomy should be determined based on the pathological diagnosis.

(Evidence level: High; Recommendation level: strong recommendation; Level of agreement: 91%).

Duodenal papillary adenoma is relatively rare clinically. Its incidence rate is less than 1 in 100,000, accounting for approximately 0.6%–0.8% of DTTs. It is commonly found in individuals aged 50 to 70 years old, and it is more prevalent in men (the male-to-female ratio is 1.5: 1).^[58,59]^ As a precancerous lesion, although duodenal adenoma is a benign disease, it follows the adenoma-adenocarcinoma pathway and has a certain risk of becoming cancerous (30%).^[60]^ Per-oral SMIS for duodenal papilla (SMIS-DP) or endoscopic papillectomy (EP) is the preferred treatment method for duodenal papillary adenoma without involvement of the pancreaticobiliary ducts. Its en bloc resection rate and radical resection rate are approximately 82.4% and 87.1% respectively, and it is particularly suitable for adenomas ≤ 3 cm. ^[61, 62, 63]^ Given the low risk of LNM, in patients with duodenal papillary adenocarcinoma confined to the mucosal layer and without local lymph node or distant metastasis (pTis and pT1a), endoscopic resection can be considered in cases in which surgical resection is not suitable or the patient refuses surgical resection.^[63,64]^ Compared with organ resection, anatomical reconstruction papillectomy, endoscopic SMIR has the advantages of less trauma, a faster recovery, and a lower incidence of adverse events. Moreover, there is no significant difference between the two methods in terms of long-term curative effect or recurrence rate.^[65,66]^

After endoscopic resection, duodenoscopy [with biopsy) should be performed for reexamination and monitoring at 3, 6, and 12 months postoperatively, and then yearly (for at least 5 years) to prevent and detect recurrence.^[62, 67]^ If the specimen after resection indicates non-R0 resection, in consideration of the patient’s general condition, characteristics of the lesion, local technical conditions, and with full informed consent, pancreaticoduodenectomy, endoscopic re-resection, radiofrequency/APC ablation, or other treatments should be performed.^[62,66,68,69]^

## Supplementary Information

Supplementary materials are only available at the official site of the journal (www.intern-med.com).

## Supplementary Material

Supplementary Material Details
