# Supplementary material for: Chinese clinical practice guidelines for super minimally invasive surgery of digestive tract tumors
Source: J Transl Int Med. 2025 Dec 22;13(6):487–507. doi: 10.1515/jtim-2025-0095 (PMC12721367; doi:10.1515/jtim-2025-0095)
Supplement: Supplementary file 1 — Supplementary Material Details [file jtim-2025-0095_sm.pdf]

Supplementary Materials

Appendix A

Analysis of relevant literature 1: The definition of SMIS was proposed by Professor Enqiang Linghu in 2016 and subsequently published in both Chinese and English journals, including *Chin Med J* (Engl), *World J Gastroenterol*, and the *Chinese Journal of Gastrointestinal Endoscopy*.[1-6] Numerous clinical studies have also confirmed that, compared to traditional open or extensive resection surgeries, SMIS significantly reduces surgical trauma, preserves organ function, lowers the risk of complications, and comprehensively enhances the postoperative physiological functions, psychological health, and social adaptability of patients, in addition to ensuring effective tumor treatment.[7-9] A single-center propensity score-matched study showed that SMIS had the same curative effect as traditional esophagectomy for the treatment of superficial esophageal squamous cell carcinoma. However, the SMIS group had significantly shorter operative times and hospital stays, less intraoperative blood loss, and a lower incidence of postoperative complications (such as pulmonary infection and anastomotic leakage) compared to the esophagectomy group.[7] Six months post-surgery, the SMIS group had significantly better swallowing function, nutritional status, and overall quality of life (as measured by the EORTC QLQ-C30 scale) than the traditional esophagectomy group, particularly in terms of preserving the esophageal physiological structure and reducing reflux symptoms.[7] Another single-center propensity score-matched study evaluated the impact of SMIS and proximal gastrectomy on the health-related quality of life in patients with early esophagogastric junction adenocarcinoma, which found that the SMIS group retained better gastric volume, had a 50% lower incidence of reflux esophagitis, and experienced significantly less postoperative weight loss than the traditional surgery group. Twelve months post-surgery, the SMIS group scored significantly better than the proximal gastrectomy group in physical function, social role functioning, and emotional health (as measured by the SF-36 scale), and had fewer dietary restrictions. The study concluded that SMIS effectively maintained digestive function by removing lesions while preserving organs, which significantly improved postoperative quality of life and psychological

adaptability.[10] A cross-sectional study comparing the effects of organ-preserving surgery and organ resection on bowel function, psychological health, and quality of life in patients with ECRC showed that the SMIS group had significantly better postoperative bowel frequency and fecal incontinence control (Wexner score), with a 60% reduction in the incidence of bowel dysfunction. The SMIS group also had significantly lower levels of anxiety and depression (as measured using the HADS scale), higher satisfaction with postoperative appearance and autonomy, and a better overall quality of life, with higher scores in physical health, social relationships, and psychological health dimensions (as measured by the WHOQOL-BREF scale) compared to the resection group(8). SMIS, while providing radical treatment of early colorectal tumors, was also concluded to maximally preserve organ function and significantly improve postoperative physical and psychological health, rendering it the preferred option for enhancing quality of life.

Analysis of relevant literature 2: A review published in the *Chinese Journal of Digestive Endoscopy* in 2021 first clarified that digestive endoscopic SMIS was implemented through four types of channels using dozens of methods, and detailed the surgical techniques and methods using different channels (including hemostasis, traction, and closure).[11] Two case reports demonstrated that the through-the-scope twin clip closure method based on SMIS effectively closed large digestive tract defects.[12, 13] One case report described the SMIS-based method of "simultaneous incision and conventional tissue clip closure" for the immediate closure of perforated defects during surgery, which effectively prevented gas and fluids from entering the abdominal cavity, and thus avoided abdominal infections.[14] A single-center retrospective study showed that the use of rubber band traction methods accelerated the efficiency of SMIS in non-FTR of colorectal lesions and assisted in closing large defects.[15] Another single-center retrospective study indicated that the tissue clip-dental floss adjustable angle traction method enhanced the efficiency of SMIS for non-FTR of gastric lesions and effectively prevented perforation complications.[16]

Analysis of relevant literature 3: In 2024, the principles for the implementation of digestive endoscopy-based SMIS were explicitly outlined in the *Chinese Journal of*

Medical Sciences, providing a scientific framework for the safe and reliable conduct of SMIS.[17] In 2023, the "Expert Consensus on Wound Preparation and Antibiotic Application for Digestive Endoscopy Super Minimally Invasive Surgery" consensus statement[18] was published, followed by an expert interpretation of its contents in the same year. This consensus offers guidance for perioperative preparation of digestive endoscopy SMIS.[19]

Analysis of relevant literature 4: The term "SMIS" was clearly defined, along with its usage and nomenclature conventions, in the book *Digestive Endoscopy Terminology*[5] published by the China National Committee for Terminology in Science and Technology in 2021. In 2023, the interpretation of the terminology of the book was examined.[20] The monograph *Digestive Endoscopy: Super Minimally Invasive Surgery*, published in 2025, uniformly applied the resulting nomenclature convention.[21] Additionally, several clinical studies have adhered to this nomenclature convention.[8,15,16,22-26]

Evidence summary 5: A systematic search and screening of 49 articles led to the inclusion of 10 relevant studies that were comprehensively analyzed. A multicenter prospective study in France (n = 195) reported a complete resection rate of 100% for early adenocarcinoma and Barrett's esophagus with HGIN treated with ESD, with an R0 resection rate of 78.5% and a curative resection rate of 67.2%. The intraoperative perforation rate was only 0.5%, but the postoperative stricture rate was 15.9%, indicating that extensive resection should be combined with preventive measures.[27] A prospective study in Denmark (n = 132) comparing ESD with EMR found that the complete resection rate (98.8% *vs*. 64.1%) and R0 resection rate (83.9% *vs*. 23.8%) were significantly higher with ESD ($P < 0.001$). Although the complication rate was higher with ESD (28.7% *vs*. 3.1%), the local recurrence rate was lower (14.5% *vs*. 23.8%).[28] A Chinese multicenter RCT (n = 40) of lesions with 50% or more circumferential involvement confirmed that ESTD was associated with a faster dissection speed ($P = 0.047$), less muscular layer damage ($P < 0.05$), and a higher mucosal healing rate one month postoperatively compared to traditional ESD.[29] A single-center prospective study in China (n = 40) comparing ESTD and ESD for early

squamous cell cancer involving 50% or more of the esophageal circumference showed that the dissection speed in the ESTD group was significantly faster than in the ESD group (P = 0.047), but there were no significant differences between the two groups in terms of operation time, dissection area, complete resection rate, R0 resection rate, or complication rate.[30] Several meta-analyses confirmed that ESD had significantly better complete resection rates, R0 resection rates, and recurrence risks than EMR, and the dissection speed of ESTD is superior to that of ESD.[9,29-33] The ASGE guidelines (2023)[34] recommend prioritizing ESD for lesions > 15 mm (evidence level: moderate) and emphasize its role in accurate staging and reducing recurrence risk, and the AGA guidelines (2020)[35] clearly identify endoscopic surgery as the preferred treatment for high-grade dysplasia or mucosal cancer in Barrett's esophagus and suggest it as an alternative to esophagectomy for low-risk T1b lesions. Additionally, a Brazilian meta-analysis incorporating 15 observational studies (as of July 28, 2023, with 9352 patients, including 2537 in the ESD group and 6,815 in the EMR group) found that the ESD group had significantly higher complete resection rates (OR = 25.96) and R0 resection rates (OR = 5.10), with a lower recurrence rate (3.08% *vs*. 11.69%, OR = 0.22).[36]

Evidence summary 6: Through a systematic search and selection of 49 articles, 7 relevant studies were ultimately used in a comprehensive analysis. A Chinese single-center prospective study (n = 81) showed that oral corticosteroids alone or combined with local corticosteroid injection effectively prevented post-ESD esophageal stricture and reduced the number of post-operative dilations, with the combination therapy more effective.[37] A Chinese single-center prospective study (n = 70) demonstrated that a combination of polyethylene glycol and stent placement more effectively prevented post-ESD esophageal stricture in patients with EEC than stent placement alone.[38] Another Chinese single-center prospective study (n = 63) showed that an aluminum phosphate gel was significantly more effective than triamcinolone acetonide injection with oral prednisone in preventing post-ESD esophageal stricture when the circumferential mucosal defect was greater than 75%, with a stricture rate of 9.4% *versus* 35.5% (best cut-off values: 97.9% *vs*. 90.6%).[39] Furthermore, a Chinese prospective study (n = 28) demonstrated that esophageal stent placement was a safe and

effective method for preventing post-ESD esophageal stricture, particularly for patients with mucosal defects exceeding 75% of the esophageal circumference.[40] Two meta-analyses from China, both showing positive results, focused on strategies to prevent post-ESD esophageal stricture in patients with EEC.[41,42]

Evidence summary 7: Through a systematic search and screening of 191 articles, ultimately 56 relevant studies were selected for comprehensive analysis. Sixteen single-center prospective studies from China and South Korea demonstrated that a comprehensive evaluation of the postoperative observation indicators reported in the literature for EGC after surgery revealed no significant difference between endoscopic surgery and “organ resection, reconstructive” surgery in terms of overall patient survival. However, endoscopic surgery was superior regarding rates of curative resection, postoperative complications, and postoperative quality of life.[43-58] Fifteen meta-analyses confirmed that there was no significant difference between endoscopic surgery and “organ resection, reconstructive” surgery in terms of overall patient survival. However, endoscopic surgery has demonstrated a significant advantage in the incidence of postoperative complications and postoperative quality of life, although it is not superior to “organ resection, reconstructive” surgery regarding the rate of curative resection.[32,59-72] Multiple retrospective studies from research centers in China, South Korea, Japan, Lithuania, and Rome confirmed there was no significant difference between endoscopic surgery and “organ resection, reconstructive” surgery in terms of overall patient survival. Endoscopic surgery was superior regarding the incidence of postoperative complications and postoperative quality of life, and was not inferior to “organ resection, reconstructive” surgery in terms of the rate of curative resection.[73-85] The American Society for Gastrointestinal Endoscopy (ASGE) clearly states in the Summary and Recommendations section of its guideline “Endoscopic Submucosal Dissection for the Management of Early Esophageal and Gastric Cancers”[34] that surgical resection is not required for EGC lesions that are well or moderately differentiated, are of an intestinal type, and have a maximum diameter of 3 cm. The guidelines[86] from the British Society of Gastroenterology regarding the diagnosis and management of patients at risk for GC suggest that all gastric mucosal dysplasia and EGC should undergo en bloc resection. EMR can achieve en bloc resection of lesions

≤ 10 mm, but only ESD can ensure en bloc resection of lesions > 10 mm. (Evidence level: High quality; Recommendation level: Strong; Agreement level: 100%). According to the “Clinical Practice Guidelines for Endoscopic Resection of Early Gastrointestinal Cancer”,[87] (1) for well or moderately differentiated tubular adenocarcinomas or papillary adenocarcinomas with a tumor maximum diameter ≤ 2 cm and diagnosed as mucosal cancer without ulcer formation during endoscopy, endoscopic resection is strongly recommended (recommendation level: strong; evidence level: moderate); (2) for lesions with similar histological characteristics, endoscopic resection is recommended when any of the following conditions are met: (i) mucosal cancer with a maximum diameter > 2 cm but no ulcer, or (ii) mucosal cancer with a maximum diameter ≤ 3 cm accompanied by an ulcer (recommendation level: weak; evidence level: moderate); and (3) for poorly differentiated tubular adenocarcinomas, low-adhesion cancer, or signet-ring cell cancer, if the maximum diameter of the tumor is ≤ 2 cm, endoscopic resection may be considered if the endoscopic evaluation is mucosal cancer without accompanying ulcers (recommendation level: weak; evidence level: low). According to the European Society of Gastrointestinal Endoscopy (ESGE) guidelines,[88] (1) for gastric superficial tumor-like lesions with an extremely low risk of LNM, endoscopic resection is strongly recommended (recommendation level: strong; evidence level: high); (2) for lesions with a diameter < 15 mm and a low risk of histological progression, EMR may be considered as a treatment option; and (3) for most gastric superficial tumor-like lesions, ESGE preferentially recommends ESD as the standard treatment option (recommendation level: strong; evidence level: moderate). Furthermore, the consensus on the detection and treatment of EGC in Mexico[89] suggests that endoscopic treatment should be performed for patients whose lesions are of a size that allows complete resection of a single specimen and whose risk of LNM is extremely low (evidence quality: C1 level; recommendation level: strong, in favor; consensus: fully agree 82%, partially agree 18%). The 2021 “Japanese Gastric Cancer Treatment Guidelines (6th edition)”[90] propose considering endoscopic resection for tumors with a very low possibility of LNM and those suitable for complete resection.

Evidence summary 8: Through a systematic search and screening of 144 articles,

19 relevant studies were included for comprehensive analysis. Several meta-analyses indicate that, compared to laparoscopic D2 radical surgery for EGC, ESD treatment showed similar survival rates and recurrence-free rates. Moreover, ESD-treated patients had shorter hospital stays, faster recoveries, and lower adverse event rates, and thus avoided overtreatment.[59-61,70-72,91-93] A multicenter retrospective study in Germany suggested ESD as the standard treatment for EGC(94). A single-center retrospective study in China (n = 324) indicated that over one-third of EGC patients treated surgically may achieve a cure through ESD.[95] Two single-center retrospective studies in China suggested that patients with undifferentiated EGC can also achieve curative resection through ESD.[96,97] A single-center retrospective study in China (n = 27) highlighted the limitations of EMR and ESD in LN clearance and the significant surgical complications associated with total gastrectomy. The study proposed that a distal gastrectomy with regional LN dissection for early gastric body cancer may avoid overtreatment and excessive surgery.[98]

Evidence Summary 9: Through systematic retrieval and screening of 146 studies, a total of 2 relevant studies were included for comprehensive analysis. A review introduced the concept, methods, and application prospects of the SMIS-Cure evaluation system.[99] A single-center prospective single-arm study (n = 8) in China showed that all eight patients with EGC successfully underwent safe sft-SMIR, achieving curative resection. The SMIS-Cure degree evaluation system was applied, and the results showed that there were seven cases of SMIS-Cure A (cured) and one case of SMIS-Cure B2 (clinically cured).[100] The establishment of the SMIS-Cure evaluation system provides a systematic framework for evaluating the cure degree of SMIS surgery for EGC, which has important clinical value and offers a clear basis for medical personnel to make treatment decisions. It should be noted that the current validation of the SMIS-Cure system for GC is still at a preliminary stage. The available evidence comes from only two studies—one review article and a small prospective study with 8 cases—which corresponds to a low level of evidence. Therefore, while the concept of the SMIS-Cure system is novel and promising, its clinical utility has yet to be confirmed. Future large-scale, multicenter studies are warranted to further evaluate

and validate this system, and it should presently be considered an important direction for ongoing research.

Evidence Summary 10: Through a systematic search and screening of 144 studies, three relevant studies were included for comprehensive analysis. According to the guidelines of the American Society for Gastrointestinal Endoscopy (2023 edition), different endoscopic follow-up strategies should be adopted for digestive tract lesions at different stages. For patients with high-grade and low-grade intraepithelial neoplasia of the stomach, endoscopic monitoring is recommended every 6 months in the first 2 years after diagnosis, and then adjusted to an annual endoscopic follow-up. For patients with T1a GC, endoscopic follow-up is recommended 3 to 6 months in the first year after surgery. The endoscopic follow-up interval can be extended to 6 to 12 months in the second and third years after surgery, and then finally switched to an annual routine endoscopic follow-up. In cases of T1b with negative lateral and basal resection margins (R0 resection), endoscopic follow-up is recommended 3 to 6 months in the first year after surgery, and then annually. An imaging evaluation should also be included, with CT examinations recommended every 6 to 12 months within 3 to 5 years after surgery to rule out potential metastasis risks. According to "The Mexican consensus on the detection and treatment of EGC",[89] after endoscopic radical resection for EGC, endoscopy recheck is recommended 6 to 12 months after surgery to monitor metachronous GC (Evidence quality C1; Recommendation strength: Strong; 82% of people strongly agree, and 18% partially agree). According to the recommendations of the "Japanese Gastric Cancer Treatment Guidelines 2021 (6th edition)"[90] different management strategies should be adopted after endoscopic resection of GC at different stages. For patients in the eCure A stage, endoscopic follow-up is recommended yearly after surgery. For patients in the eCure B stage, monitoring must be strengthened, with 1 or 2 endoscopic examinations combined with abdominal ultrasound or CT evaluation recommended each year. Regardless of stage, the guidelines emphasize routine *Helicobacter pylori* testing after surgery, and if the result is positive, eradication treatment should be carried out in a timely manner. Due to the long-term risk of metachronous GC, a long-term follow-up plan should be established for all patients. In eCure C1 stage cases, if the lesion is a differentiated GC $\leq$ 3 cm and has any of the

characteristics of UL1, pT1a (M), or pT1b1 (SM1), the range of residual mucosal lesions must be re-evaluated through endoscopy. When the total length of the primary focus and the residual focus exceeds 3 cm, the guidelines clearly list gastrectomy combined with LN dissection as the standard treatment plan. For patients in the eCure C2 stage, gastrectomy combined with LN dissection is recommended as the standard treatment method.

Evidence summary 11: Through systematic retrieval and screening of 168 studies, a total of 10 relevant studies were selected for comprehensive analysis. A single-center prospective study in Japan (*n* = 300) showed that a decrease in hemoglobin of ≥ 2 g/dl was observed in 5% of ESD cases (LAC: 30.0%). Complications occurred in 7% of ESD cases (LAC: 15%). The incidences of delayed bleeding and perforation were 5 and 1.7% of ESD cases, respectively. Although only one patient required laparotomy for peritonitis caused by delayed perforation, other patients could be treated endoscopically. Due to the redefinition of the risk of LNM, 16 ESD cases required additional LAC. The median length of the hospital stay for ESD was 5 days (LAC: 10 days).[101] A multicenter prospective study in the Netherlands (*n* = 1315) indicated that additional surgery should only be considered for patients with high histological risk or positive resection margins after endoscopic resection.[102] A single-center prospective study in China (*n* = 120) showed that the operation time, recovery time of gastrointestinal function, length of hospital stay, and treatment costs of patients in the ESD group were significantly better than those in the conventional surgical resection group ($P < 0.05$), and the negative margin rate of tumors in the ESD group was significantly lower than in the conventional surgical resection group ($P < 0.05$).[103] One meta-analysis reported positive results suggesting that compared with surgery, the operation time and length of hospital stay of the ESD group were better than those of the surgical group. However, the en bloc resection rate and radical resection rate in the ESD group were lower, but there was no significant difference in postoperative complications or the recurrence rate between the two groups(104). A multicenter retrospective study in Japan (*n* = 701) showed that ER for T1 CRC did not deteriorate the clinical outcomes of patients who required additional surgical resection.[105] There were a total of four single-center retrospective studies, all of which suggested that minimally invasive endoscopic

treatment was as effective as surgery but less invasive, and that it could become the first-line treatment for ECRC.[106-109] A prospective multicenter cohort study from Japan and Greece included 23 patients treated with EID who had either deep submucosal invasive RC, scarred and severely fibrotic rectal lesions, or benign tumors. The results showed that there were no serious complications in any patients; pain was reported in four cases, transient urinary incontinence occurred in one case, postoperative stricture occurred in one case that was supplemented with dilation treatment, and incomplete resection of a T2 tumor was found in one case. The EID surgical resection plane entered the intermuscular space between the longitudinal (external) and circular (internal) muscle layers. This technique showed great potential for endoscopic radical resection of invasive rectal submucosal cancer.[110]

Evidence Summary 12: Through a systematic search and screening of 216 articles, a total of 11 relevant studies were selected for comprehensive analysis. A multicenter prospective study in the Netherlands ($n = 67$) showed that the final complete resection rate and curative resection rate of EID for colorectal tumors were 96 and 81%, respectively. The average operation time (standard deviation) was 110 ± 51 min, and the average tumor size was 25 mm. Minor adverse events occurred in 8 patients, with moderate perianal pain in three patients (4%) and inflammatory reactions (fever, pain, elevated C-reactive protein, but no fluid accumulation in the perirectal air) in 3 patients (4%). All patients were conservatively treated with intravenous antibiotics and/or analgesics.[111] In a single-center prospective study in Japan ($n = 23$), 22 of 23 patients with colorectal tumors underwent en bloc endoscopic complete resection (96%, 95% CI: 90%–99%), while the complete dissection rate of benign scar lesions was 100% (12/12), confirming that EID ensured margin-free resection, or at least provided accurate diagnostic pathological support for prospective oncological surgery of T2 tumors.[110] Multiple meta-analyses have shown that compared with EMR, ESD is associated with more postoperative complications, including perforation and bleeding, but has a higher R0 resection rate and better prognosis. Therefore, ESD is more often recommended for patients with ECRC. Compared with TEM, ESD had fewer postoperative complications, with no significant difference in the R0 resection rate.[112-116] A single-center retrospective study in China used an endoscopic and laparoscopic

combined surgery for treatment. Patients with T2 CRC underwent FTR, and peripheral LN dissection was performed. Different endoscopic treatment methods were selected according to risk stratification of ECRC to formulate a precise treatment strategy for patients.[117] A multicenter prospective study in Denmark (n = 25) treated patients with CRC (cT1-2cN0cM0) with combined endoscopic-laparoscopic surgery (CELS) and concluded that in select patients, CELS resection was feasible and avoided colectomy.[118] According to the ESGE guidelines (2024),[119] in the treatment of suspected superficial invasive cancer, if standard polypectomy or EMR cannot achieve complete resection, the following techniques are recommended as the preferred options: EMR, ESD, EID, EFTR, or surgical resection with complete resection (Evidence level: moderate). The NCCN *Clinical Practice Guidelines in Oncology*[120] indicated that compared with EMR, the main advantages of ESD are a higher en bloc resection rate and a lower recurrence rate; the disadvantages are a longer operation time, a higher complication rate, and a steep learning curve. According to the morphology of the lesion, colorectal ESD can be considered for lesions with a high suspicion of limited superficial mucosal invasion. For T2 CRC, due to the higher risk of LNM, FTR and LN dissection are recommended. A meta-analysis from China included five RCTs and eight cohort studies. The results showed that compared with radical surgery, TEM was associated with a higher risk of local recurrence, but had advantages regarding disease-free survival, reduction of perioperative mortality, temporary stoma, permanent stoma, and postoperative complications. TEM also effectively shortened the operation time, postoperative bleeding, and hospital stay. There were no statistically significant differences between the two surgical approaches in terms of the incidence of distant metastasis, overall recurrence, or disease-specific survival rates.[121] A randomized controlled trial in China compared the data of 60 patients with T1-2N0 RC who underwent TEMS and laparoscopic anterior resection (LAR). The results showed that the operation time and postoperative defecation recovery time in the TEMS group were significantly shorter than those in the LAR group, with the postoperative defecation recovery time earlier (1 day *vs*. 3 days). The complications in both groups were generally mild and self-limiting; however, two patients at T2 stage in the TEMS group (2/28, 7.1%) had local recurrences (8 months and 16 months after surgery), while there

were no recurrences in the LAR group (0/30), suggesting that although TEMS accelerated postoperative recovery and reduced surgical risks, its local control ability of T2 tumors needs to be carefully evaluated.[122]

Evidence Summary 13: Through a systematic search and screening of 225 studies, 13 relevant studies were included for a comprehensive analysis. A multicenter prospective study in Japan (*n* = 1090) showed that the final complete resection rate and curative resection rate of patients with colorectal tumors treated with ESD were 88% and 89%, respectively.[123] A meta-analysis in China compared the efficacy of ESD and EMR in the treatment of DTTs, with positive results. The study suggested that compared with EMR, ESD showed a better complete resection rate, curative resection rate, and lower local recurrence rate, but with a longer operation time and a higher incidence of bleeding and perforation complications.[124] A multicenter retrospective study in Germany (*n* = 1234) showed that the R0 resection rate of patients who received EFTR treatment due to various indications was 71.8%(125). A multicenter retrospective study in Belgium and Portugal (*n* = 252) showed that the R0 resection rate of rectal ESD cases was 75%, and the curative resection rate was 70%(126). There were a total of nine single-center retrospective studies, all with positive results, with significant differences in diseases and stages in these studies.[127-135] The 2022 version of the NCCN "Guidelines for Colorectal Cancer"[136] proposed that for malignant polyps with complete resection and good histological features, resection is usually considered curative, and no additional surgery is required. If adverse histological features are found, further examinations (such as imaging) may be needed, and surgeries such as colectomy may be required. The 2023 CSCO "Colorectal Cancer Guidelines"[137] propose a structured and layered optimization of endoscopic treatment for T1N0 stage colon cancer. Complete resection is recommended for flat lesions (diameter 5–20 mm), extensive lesions suspected to be villous adenomas or extensive serrated adenomas (> 10 mm), or suspected HGIN (⩽ 20 mm) (EMR is recommended for grade I, ESD is recommended for grade II). For some T1 stage colon cancer (submucosal infiltration < 1 mm), laterally developing tumors (LST ⩾ 20 mm), or fibrotic villous adenomas (⩾ 25 mm), ESD is recommended if grade I, and surgical treatment is recommended if grade II. To determine curative resection, the following histological criteria must be

met: submucosal infiltration depth < 1 mm, no lymphatic vessel invasion, good tumor differentiation, zero tumor budding, and negative margin (⩾ 1 mm from the margin). In terms of postoperative management, follow-up endoscopy is recommended 3–6 months after surgery for patients with unclear surgical margins, and 1 year after surgery for patients with negative surgical margins. The 2020 version of the ESMO "Guidelines for Local Colon Cancer"[138] proposed that for hyperplastic or adenomatous polyps and non-invasive adenocarcinoma (pTis, intraepithelial or intramucosal), endoscopic resection is sufficient. For invasive cancer (pT1), the treatment method depends on the polyp morphology and the presence of histological features associated with a poor prognosis. For pedunculated polyps with pT1 cancer limited to the head, neck, and stalk (Haggitt 1–3) without unfavorable factors, even if there is submucosal invasion, endoscopic resection and appropriate follow-up are sufficient. However, for patients with an average surgical risk, if sessile or flat polyps (Paris classification) are associated with pT1 cancer and there are any unfavorable factors, surgical resection must be performed. A positive resection margin (< 1 mm) only constitutes a risk of local recurrence, which can be managed with repeated resection or local monitoring. If surgery is not possible due to severe comorbidities, colonoscopic monitoring is recommended within 6 months after polypectomy, with close oncological follow-up, including CT scans to detect LN recurrence. The 2019 version of the JSCCR "Guidelines for Colorectal Cancer"[139] proposed that for piecemeal resection of sheet-like pTis cancer with positive horizontal margins, local recurrence should be checked by colonoscopy around 6 months. In the follow-up observation of pT1 cancer, local recurrence, LN recurrence, and distant metastasis recurrence should be evaluated. The follow-up should include endoscopic examination, imaging diagnosis, and tumor marker evaluation. According to "Expert Consensus on Management Strategies for Precancerous Lesions and Conditions of Colorectal Cancer in China"[140] the preferred method for endoscopic resection of early CRC and HGIN is ESD. In cases with positive vertical margins in postoperative pathology, additional treatment should be considered after confirming residual lesions. If the lesion has been completely removed and meets the following five conditions simultaneously, it can be considered as achieving curative resection: negative vertical margin; pathological type of papillary adenocarcinoma or

tubular adenocarcinoma; submucosal infiltration depth < 1000 μm; no vascular invasion; and tumor budding is grade 1 (low grade). If any of the above five conditions are not met, a comprehensive assessment of the risk of LNM and the specific situation of the patient (including age, underlying diseases, physical condition, personal wishes, and postoperative quality of life) is required to determine whether additional surgery is needed. In addition, in cases with positive vertical margins due to technical limitations, after a detailed evaluation, endoscopic resection may be considered again. An expert consensus on endoscopic diagnosis and treatment of CRC and precancerous lesions in China (2023, Guangzhou)[141] proposed that if the postoperative pathology report indicates that the epithelial tumor has not penetrated the muscularis mucosae and invaded the submucosa, and the resection margin is negative, it is regarded as a complete and curative resection, and no additional treatment is required .

Evidence summary 14: Through a systematic search and screening of 145 articles, 12 relevant studies were selected for comprehensive analysis. A prospective study involving 362 patients with locally advanced non-metastatic RC (≥ T3 or Tany N+ any without distant metastasis) showed that there were no significant differences in the overall survival rate (85.6% *vs*. 93.3%, $P = 0.414$) or disease-free survival rate (78.3% *vs*. 80%, $P = 0.846$) between radical surgery and LE.[142] A multicenter prospective study in France showed that among 74 patients in the LE group, 26 patients subsequently underwent TME surgery, and it could not be confirmed that LE was superior to TME.[143] A multicenter prospective study in the Netherlands included 55 RC patients with an expecting treatment response after long-course concurrent chemoradiotherapy. Eventually, 47 patients received TEM, with a 5-year overall survival rate of 82.8%.[144] A prospective cohort study in the Netherlands included 67 patients with suspected deep submucosal invasive RC who underwent EID. The overall technical success rate of the treatment was 96%, the R0 resection rate was 81%, and the radical resection rate was 45%.[111] A meta-analysis in China included four retrospective cohort studies and seven prospective non-randomized controlled trials (a total of 1131 patients). By comparing the effectiveness and safety of the W&W strategy, radical surgery, and LE in RC patients with a cCR after neoadjuvant chemoradiotherapy, it was found that the local recurrence rate in the W&W group was higher than in the

radical surgery group, and there were no significant differences in distant metastasis and mortality rates among the three groups of patients.[145] Two Chinese case series studies included patients who achieved cCR or near-CR after neoadjuvant chemoradiotherapy and LE, but experienced local recurrence and distant metastasis upon follow-up. Both studies suggested that for RC patients evaluated as having cCR after receiving neoadjuvant chemoradiotherapy and undergoing LE due to a strong desire to preserve the anus, LE has certain diagnostic and therapeutic value, but the indications must be strictly selected.[146,147] A retrospective study compared the prognosis of RC patients who achieved cCR after chemoradiotherapy and received radical surgery (37 patients) and those who received only LE (14 patients). The disease-free survival rate, pelvic recurrence-free survival rate, and overall survival rate of the two groups of patients were similar.[148] A Chinese retrospective cohort study compared the efficacy of TEM and transanal total mesorectal excision (taTME) and found that the 5-year progression-free survival rates of the two groups were similar.[149] “Expert Consensus on the Early Diagnosis and Treatment of Colorectal Cancer in China” (2023 edition)[150] recommends intersphincteric resection (ISR) as an option for ultra-low anal-preserving surgery for patients with cT1 stage ultra-low and low RC not suitable for LE, and for some RC patients whose cancer was downstaged after preoperative chemoradiotherapy. For cT1N0M0 RC patients with high-risk factors for metastasis and a strong desire to preserve the anus, concurrent chemoradiotherapy combined with LE (including TEM and TAMIS) can be adopted (Recommendation level: Strong; GRADE classification: moderate). The ESMO “Guidelines for Rectal Cancer”[151] recommend that for low cT3a and cT3b, medium and high cT3a and cT3b, and cN1–2 RC with negative mesorectal fascia and negative extramural vascular invasion, TME surgery can be directly performed; the treatment strategy for the remaining LARC is the “sandwich treatment” of preoperative concurrent chemoradiotherapy, TME surgery, and postoperative adjuvant chemotherapy. The “Chinese Expert Consensus on the Watch-and-Wait Strategy after Neoadjuvant Therapy for Rectal Cancer” (2024 edition)[152] recommends that for patients with a stage of ycT1N0M0 after neoadjuvant therapy, patients in which there is difficulty preserving the anus, and patients with a strong desire to preserve the anus, LE can be selected. (Evidence level: III,

Recommendation level: B level, Expert group approval rate: 96.77%). A meta-analysis in the UK included 17 studies, of which 10 were retrospective cohort studies and 4 were prospective studies. The results showed that the 2-year local recurrence rate using the W&W strategy was 15.7%, the 3-year local recurrence rate was approximately 22%, and the vast majority of patients chose supplementary surgery after local recurrence.[153,154] A retrospective observational study in the United States (*n* = 249) analyzed and compared the W&W strategy of patients who achieved cCR after completing neoadjuvant therapy and in rectal adenocarcinoma patients who received TME and achieved a pCR. The results showed that the 5-year disease-free survival rate, overall survival rate, and disease-specific survival rate in the W&W group were all inferior to those of TME. Furthermore, in the W&W group, the survival rate of patients with local recurrence was poorer, and the incidence of distant progression was higher.[155]

Evidence summary 15: A meta-analysis that included 29 studies with a total of 1,751 patients indicated that for the treatment of ampullary neoplastic lesions by SMIS-EP, the en bloc resection rate was 82.4% (95% CI: 74.7%–88.1%), and the radical resection rate was 87.1% (95% CI: 83.0%–90.3%). The overall incidence of adverse events was 24.9% (95% CI: 21.2%–29.0%), among which postoperative pancreatitis was the most common, accounting for approximately 11.9% (95% CI: 10.4%–13.6%).[156] A retrospective study published in *Gut* in 2025,[157] which involved 1673 patients from 58 centers in Europe and the United States, showed that by treating ampullary adenoma by endoscopic resection, the R0 resection rate was 80.2% (386/481), the incidence of severe adverse events was 1.9% (11/569), there were no deaths, and the average follow-up period was 20 months (ranging from 9 to 46 months), with a recurrence rate of approximately 13.5% (65/481). After propensity score matching, the R0 resection rates of endoscopic resection and surgical papillectomy were 72.6% and 90.3% respectively ($P = 0.02$), with no significant difference in the recurrence rate (8% *vs*. 3.2%, $P = 0.07$) or survival rate between the two groups. However, there was one death in the surgical group, and the operation time and hospital stay in the endoscopic group were significantly shorter than those of the surgical group (36 min *vs*. 225 min, 3 days *vs*. 14 days, $P < 0.001$). Postoperative recurrence is a matter

of widespread concern. In 2023, Garg *et al*.[158] published a meta-analysis (39 studies with 1753 patients) comparing endoscopic resection with surgical resection to evaluate postoperative recurrence. The 1-, 2-, 3-, and 5-year recurrence rates of endoscopic resection (13.0%, 12.5%, 13.3%, and 15.7%, respectively) and surgical resection (14.1%, 14.3%, 12.9%, and 17.6%, respectively) showed no significant difference ($P > 0.05$). Internationally, guidelines developed by multiple societies such as those in Europe, France, and Japan (*e.g.*, ESGE, JGES) all recommend endoscopic papillectomy as the treatment of choice for duodenal papillary adenoma.[159-161]

References:

1. Linghu E. A new stage in the history of surgery: super minimally invasive technique. Chin J Gastrointest Endosc (Electron Ed) 2016;3:97-98.
2. Linghu E. New direction for surgery: Super minimally invasive surgery. World J Gastroenterol 2024;30:1676-1679.
3. Linghu E. Three stages of the development of gastrointestinal endoscopic therapeutics. Chin J Gastrointest Endosc (Electron Ed) 2024;11:73-74.
4. Linghu E. A new stage of surgical treatment: super minimally invasive surgery. Chin Med J (Engl) 2022;135:1-3.
5. Linghu E. Nomenclature of Super Minimally Invasive Surgery: Interpretation of Nomenclature of Digestive Endoscopy by the National Committee for Terms in Sciences and Technologies. Chin J Gastrointest Endosc (Electron Ed) 2023;10:3.
6. Linghu E. Surgery should allow time for new science to unlock the human body's vast potential-The division and reflection of surgical eras. Chin J Gastrointest Endosc (Electron Ed) 2025;12:1-2.
7. Feng J, Chai N, Linghu E, Feng X, Li L, Du C, *et al*. Outcomes of super minimally invasive surgery vs. esophagectomy for superficial esophageal squamous cell carcinoma: a single-center study based on propensity score matching. Chin Med J (Engl) 2022;135:2491-2493.
8. Cai Z, Yuan X, Li H, Feng X, Du C, Han K, *et al*. Bowel function, quality of life, and mental health of patients with high-grade intraepithelial neoplasia or T1 colorectal cancer after organ-preserving versus organ-resection surgeries: a cross-sectional study at a Chinese tertiary care center. Surg Endosc 2024;38:5756-5768.
9. Liu S, Chai N, Lu Z, Li H, Xiong Y, Zhai Y, *et al*. Long-term outcomes of superficial neoplasia at the esophagogastric junction treated via endoscopic submucosal dissection and endoscopic submucosal tunnel dissection: a cohort study of a single center from China. Surg Endosc 2020;34:216-225.
10. Meng M, Chai N, Liu S, Feng X, Linghu E. Health-related quality of life after super minimally invasive surgery and proximal gastrectomy for early-stage adenocarcinoma of the esophagogastric junction: a propensity score-matched study. Chin Med J (Engl) 2022;135:3022-3023.

11. Ning B, Linghu E. New advances in different treatment channels of super minimally invasive surgery by digestive endoscopy. Chin J Dig Endosc 2021;38:939-973.
12. Li C, Chai N, Wang T, Li S, Huang F, Zhang N, *et al*. Application of through-the-scope twin clip for semicircumferential defect closure after rectal laterally spreading tumor transanal super minimally invasive surgery resection. Endoscopy 2024;56:E963-e4.
13. Li C, Li S, Wang T, Xu Y, Xu Z, Huang F, *et al*. Application of through-the-scope twin clip for defect closure after gastric gastrointestinal stromal tumor transoral super minimally invasive surgery resection. Endoscopy 2024;56:E603-e4.
14. Li X, Zhang W, Gao F, Dong H, Wang J, Chai N, *et al*. A modified endoscopic full-thickness resection for gastrointestinal stromal tumors: A new closure technique based on the instruction of super minimally invasive surgery. Endoscopy 2023;55:E561-e2.
15. Cai ZN, B., Shao Q, Chen Q. Application and surgical techniques of rubber band traction in super minimally invasive surgery of colorectal lesions. Chin J Postgrad Med 2024;47:301-305.
16. Shao Q, Wang X, Han K, Li J, Cai Z, Linghu E, *et al*. Application of` the variable angle traction method by using clip with dental in super minimally invasive non full thickness resection of early gastric cancer. Chin J Postgrad Med 2024;47:221-225.
17. Chen Q, Yuan Y, Linghu E. Advances in the diagnosis and treatment of gastrointestinal tumors under the concept of super minimally invasive surgery. J Transl Int Med 2025;13:183-186.
18. Endoscopology CSoD. Chinese expert consensus on wound pretreatment and antibiotic application in endoscopic super minimally invasive surgery(2023, Beijing). Chin J Gastrointest Endosc (Electron Ed) 2023;10:83-91.
19. Gao F, Chen Q, Linghu E. Interpretation of Chines expert consensus on wound pretreatment and antibiotic application in endoscopic super minimally invasive surger (2023, Beijing). Chin J Gastrointest Endosc (Electron Ed) 2023;10:145-147.
20. Linghu E. Chinese Terms in Digestive Endoscopology. Beijing: China National Committee for Terminology in Science and Technology; 2021.
21. Linghu E. Digestive Endoscopy: Super Minimally Invasive Surgery. Science Press; 2025. Accessed December 1, 2025.
22. Cai Z, Chen Q, Ning B, Li H, Yuan X, Linghu E. Changes in quality of life and psychological status of patients with colorectal cancer under the super minimally invasive surgery mode and the organ resection mode. Chin J Gastrointest Endosc (Electron Ed) 2024;11:81-87.
23. Wang L, Lyu K, Li Y, Chen Q. Feasibility analysis of digestive endoscopic super minimally invasive surgery of non-ampullary duodenal lesions. Chin J Postgrad Med 2024;47:296-300.
24. Wang J, Sun Y, Li W, Xiao P, Chen Q. Clinical analytical results of enhanced abdominal CT detection of lymph node metastasis in patients with non-curable resection of early gastric cancer to guide the rational treatment of super minimally invasive surgery. Chin J Postgrad Med 2024;47:306-309.
25. Sun G, Chen Q, Wang L, Lyu K, Dong X, Shi H. Therapeutic efficacy assessment of super minimally invasive surgery in the treatment of early colorectal cancer and precancerous lesions. Chin J Postgrad Med 2024;47:292-296.
26. He SD, Zhang SQ, Ge CY, Zhang L, Chen QQ, Teng GG. [Clinical characteristics of appendix orifice polyps and the effect endoscopic super minimally invasive treatment]. Zhonghua Yi Xue Za Zhi 2024;104:3342-3346.
27. Stephant S, Jacques J, Brochard C, Legros R, Lepetit H, Barret M, *et al*. High proficiency of

esophageal endoscopic submucosal dissection with a "tunnel + clip traction" strategy: a large French multicentric study. Surg Endosc 2023;37:2359-2366.

28. Schæbel GH, Mucha AW, Egeland C, Achiam MP. Endoscopic submucosal dissection and endoscopic mucosal resection for esophageal and gastric lesions: A comparison of procedures. Laparoendosc Endosc Robot Surg 2024;7:66-71.

29. Fan X, Wu Q, Li R, Chen W, Xie H, Zhao X, *et al*. Clinical benefit of tunnel endoscopic submucosal dissection for esophageal squamous cancer: a multicenter, randomized controlled trial. Gastrointest Endosc 2022;96:436-44.

30. Li Y, Wang K, Shi Y, Zhu J, Cui R, Zhang H, *et al*. Comparison of Short-Term Efficacy Between Endoscopic Submucosal Tunnel Dissection and Endoscopic Submucosal Dissection in Treatment of Wide Esophageal Squamous Cell Carcinoma of Early Stage. J Clin Gastroenterol 2020;54:512-516.

31. Dan X, Lv XH, San ZJ, Geng S, Wang YQ, Li SH, *et al*. Efficacy and Safety of Multiband Mucosectomy Versus Cap-assisted Endoscopic Resection For Early Esophageal Cancer and Precancerous Lesions: A Systematic Review and Meta-Analysis. Surg Laparosc Endosc Percutan Tech 2019;29:313-320.

32. Wang HY, Zeng X, Bai SY, Pu K, Zheng Y, Ji R, *et al*. The safety and efficacy of endoscopic submucosal dissection for treating early oesophageal carcinoma: a meta-analysis. Ann R Coll Surg Engl 2020;102:702-711.

33. Zhang W, Zhai Y, Chai N, Linghu E, Lu Z, Li H, *et al*. Endoscopic submucosal tunnel dissection and endoscopic submucosal dissection for large superficial esophageal squamous cell neoplasm: efficacy and safety study to guide future practice. Surg Endosc 2018;32:2814-2821.

34. Forbes N, Elhanafi SE, Al-Haddad MA, Thosani NC, Draganov PV, Othman MO, *et al*. American Society for Gastrointestinal Endoscopy guideline on endoscopic submucosal dissection for the management of early esophageal and gastric cancers: summary and recommendations. Gastrointest Endosc 2023;98:271-284.

35. Sharma P, Shaheen NJ, Katzka D, Bergman J. AGA Clinical Practice Update on Endoscopic Treatment of Barrett's Esophagus With Dysplasia and/or Early Cancer: Expert Review. Gastroenterology 2020;158:760-769.

36. Gallegos MMM, Gomes ILC, Brunaldi VO, Bestetti AM, Marques SB, Miyajima NT, *et al*. Endoscopic submucosal dissection vs. endoscopic mucosal resection in the treatment of early Barrett's neoplasia: Systematic review and meta-analysis. Dig Endosc 2024;36:1299-1311.

37. Wu H, Chen L, Wei M, Lu Q, Li N, Wang G, *et al*. Effect and mechanism of glucocorticoids in preventing stenosis after esophageal endoscopic submucosal dissection. Chin Med J 2022;102:1506-1511.

38. Chai NL, Feng J, Li LS, Liu SZ, Du C, Zhang Q, *et al*. Effect of polyglycolic acid sheet plus esophageal stent placement in preventing esophageal stricture after endoscopic submucosal dissection in patients with early-stage esophageal cancer: A randomized, controlled trial. World J Gastroenterol 2018;24:1046-1055.

39. Zhang Y, Yan X, Huang Y, Nie D, Wang Y, Chang H, *et al*. Efficacy of oral steroid gel in preventing esophageal stricture after extensive endoscopic submucosal dissection: a randomized controlled trial. Surg Endosc 2022;36:402-412.

40. Wen J, Lu Z, Yang Y, Liu Q, Yang J, Wang S, *et al*. Preventing stricture formation by covered esophageal stent placement after endoscopic submucosal dissection for early esophageal cancer.

Dig Dis Sci 2014;59:658-663.
41. Zhang BZ, Zhang Y, Wang YD, Liao Y, Zhang JJ, Wu YF, *et al*. Stent placement to prevent strictures after esophageal endoscopic submucosal dissection: a systematic review and meta-analysis. Dis Esophagus 2021;34:doab015.
42. Yu JP, Liu YJ, Tao YL, Ruan RW, Cui Z, Zhu SW, *et al*. Prevention of Esophageal Stricture After Endoscopic Submucosal Dissection: A Systematic Review. World J Surg 2015;39:2955-2964.
43. Li X, Qi H, Duan S, Shen F, Zhao C, Yang Y. Clinical effects of endoscopic submucosal dissection and traditional surgery in early gastric cancer. Minerva Gastroenterol (Torino) 2024;70:131-133.
44. Pyo JH, Lee H, Min BH, Lee JH, Choi MG, Lee JH, *et al*. Long-Term Outcome of Endoscopic Resection vs. Surgery for Early Gastric Cancer: A Non-inferiority-Matched Cohort Study. Am J Gastroenterol 2016;111:240-249.
45. Chiu PWYW, Teoh AY, Ng EK, Wong VW, Yip HC, Wu JC, *et al*. 375 Endoscopic Submucosal Dissection Compared to Laparoscopic Gastrectomy for Treatment of Early Gastric Cancer and a Prospective Randomized Trial. Gastrointest Endosc 2017;85:AB70-AB71.
46. Kim YI, Kim YA, Kim CG, Ryu KW, Kim YW, Sim JA, *et al*. Serial intermediate-term quality of life comparison after endoscopic submucosal dissection versus surgery in early gastric cancer patients. Surg Endosc 2018;32:2114-2122.
47. Zhou H, Wang Y, Yu W. The Comparative Study of Laparoscopic Radical Resection for Gastric Cancer and Endoscopic Submucosal Dissection in the Treatment of Early Gastric Cancer. Acta Acad Med Weifang 2017;39:440-442.
48. Sun R. Analysis of Clinical Efficacy of Endoscopic Submucosal Dissection and Radical Gastrectomy in the Treatment of Early Gastric Cancer. China Pract Med 2019;14:53-54.
49. Xie D. Clinical efficacy analysis of endoscopic submucosal dissection for patients with early gastric carcinoma. China Pract Med 2018;13:1-3.
50. Wang Z, Li J, Zhang Q, Duan T, Kang Q. Efficacy of Endoscopic Submucosal Dissection versus Totally Laparoscopic Surgery in the Treatment of Elderly Patients with Early Gastric Cancer and Their Impacts on Complications and Short-Term Prognosis. Chin J Gerontol 2023;43:2610-2613.
51. Chen M, Wu K, Lin J. Comparison of the effect and occurrence of complications of endoscopic submucosal dissection and conventional surgery for early gastric cancer. Chin J Modern Drug Appl 2018;2:14-16.
52. He S. Comparison of Efficacy between Endoscopic Submucosal Dissection and Open Surgery in the Treatment of Early Gastric Cancer. Contemp Med Forum 2017;15:94-95.
53. Xie X, Shen J, Li J, Cheng S. Comparison of efficacy between endoscopic submucosal dis-section and laparoscopic surgery for early gastric cancer. Chinese J Curr Adv Gen Surg 2019;22:935-937,944.
54. Chen M, Wu K, Lin J. Comparison of the effect and occurrence of complications of endoscopic submucosal dissection and conventional surgery for early gastric cancer. Chinese J Modern Drug Appl 2018;12:14-16.
55. Yang Y, Luo H, Ding X, Gao X. Study on the Difference of Curative Effect Between Endoscopic Submucosal Dissection and Surgery in the Treatment of Early Gastric Cancer. China Med Device Inf 2021;27:81-83.
56. Hu H, Sun X, Zhou Q, Ye J. Comparison of Clinical Efficacy and Quality of Life Between Endoscopic Submucosal Dissection and Surgical Treatment for Early Gastric Cancer. Pract J Cancer

2023;38:1165-1168.
57. Gao Y, Hu L. Clinical Efficacy of Endoscopic Submucosal Dissection versus Surgical Surgery in the Treatment of Early Gastric Cancer and Its Impact on Patient Satisfaction. Chinese J Med Res 2019;17:115-117.
58. Yang M, Li H, Zhao M. Comparative Analysis of Clinical Efficacy between Endoscopic Submucosal Dissection (ESD) and Traditional Surgery in the Treatment of Patients with Early Gastric Cancer. Chinese J Gerontol 2019;39:2105-2107.
59. Kondo A, de Moura EG, Bernardo WM, Yagi OK, de Moura DT, de Moura ET, *et al*. Endoscopy vs surgery in the treatment of early gastric cancer: Systematic review. World J Gastroenterol 2015;21:13177-13187.
60. Bestetti AM, de Moura DTH, Proença IM, Junior E, Ribeiro IB, Sasso J, *et al*. Endoscopic Resection Versus Surgery in the Treatment of Early Gastric Cancer: A Systematic Review and Meta-Analysis. Front Oncol 2022;12:939244.
61. Liu Q, Ding L, Qiu X, Meng F. Updated evaluation of endoscopic submucosal dissection versus surgery for early gastric cancer: A systematic review and meta-analysis. Int J Surg 2020;73:28-41.
62. Sun K, Chen S, Ye J, Wu H, Peng J, He Y, *et al*. Endoscopic resection versus surgery for early gastric cancer: a systematic review and meta-analysis. Dig Endosc 2016;28:513-525.
63. Yang HJ, Kim JH, Kim NW, Choi IJ. Comparison of long-term outcomes of endoscopic submucosal dissection and surgery for undifferentiated-type early gastric cancer meeting the expanded criteria: a systematic review and meta-analysis. Surg Endosc 2022;36:3686-3697.
64. Benites-Goñi H, Palacios-Salas F, Carlin-Ronquillo A, Díaz-Arocutipa C, Piscoya A, Hernández A. Endoscopic submucosal dissection versus surgery for patients with undifferentiated early gastric cancer. Rev Esp Enferm Dig 2023;115:3-9.
65. Zhao S, Zhang X, Wang J, Ge J, Liu J. Endoscopic resection versus surgery for early gastric cancer and precancerous lesions: a meta-analysis. Springerplus 2016;5:678.
66. Huh CW, Ma DW, Kim BW, Kim JS, Lee SJ. Endoscopic Submucosal Dissection versus Surgery for Undifferentiated-Type Early Gastric Cancer: A Systematic Review and Meta-Analysis. Clin Endosc 2021;54:202-210.
67. Gu L, Khadaroo PA, Chen L, Li X, Zhu H, Zhong X, *et al*. Comparison of Long-Term Outcomes of Endoscopic Submucosal Dissection and Surgery for Early Gastric Cancer: a Systematic Review and Meta-analysis. J Gastrointest Surg 2019;23:1493-1501.
68. Wu D. Meta-Analysis of Efficacy Comparing Endoscopic Submucosal Dissection versus Surgical Surgery in the Treatment of Early Gastric Cancer. Nanchang, China: Nanchang University; 2022.
69. Wang Z. Comparison of Quality of Life and Prognosis in Patients with Early Gastric Cancer Treated by Endoscopic Submucosal Dissection versus Surgical Surgery: A Systematic Review and Meta-Analysis. Shenyang, China: China Medical University; 2022.
70. Zhong C, Yang J, Li R, Wang Q, Chen Y, Li G. Endoscopic submucosal dissection and gastrectomy for early gastric cancer: a Meta-Analysis. Chin J Endosc 2017;23:57-63.
71. Dong Y, Dang Y, Zhang G. The efficacy and safety of endoscopic submucosal dissection and surgery in treat-ment of early gastric cancer: a Meta-analysis. Chin J Gastroenterol Hepatol 2018;27:1223-1227.
72. Zhu X, Ma R, Yu H. Efficacy of endoscopic submucosal dissection and surgery for early gastric cancer in China:A Meta-Analysis. Chin J Cancer Prev Treat 2019;26:1296-1305.

73. Liu Y, Chen Z, Zhou H, Chen Y, Dou L, Zhang Y, *et al*. Comparison of Endoscopic Submucosal Dissection and Radical Surgery for Early Gastric Cancer in Remnant Stomach. J Clin Med 2022;11:5403.
74. Ahn JY, Kim YI, Shin WG, Yang HJ, Nam SY, Min BH, *et al*. Comparison between endoscopic submucosal resection and surgery for the curative resection of undifferentiated-type early gastric cancer within expanded indications: a nationwide multi-center study. Gastric Cancer 2021;24:731-743.
75. Tae CH, Shim KN, Kim BW, Kim JH, Hong SJ, Baik GH, *et al*. Comparison of subjective quality of life after endoscopic submucosal resection or surgery for early gastric cancer. Sci Rep 2020;10:6680.
76. Bausys R, Bausys A, Stanaitis J, Vysniauskaite I, Maneikis K, Bausys B, *et al*. Propensity score-matched comparison of short-term and long-term outcomes between endoscopic submucosal dissection and surgery for treatment of early gastric cancer in a Western setting. Surg Endosc 2019;33:3228-3237.
77. Zhou P, Peng G, Yang S, Chen D, Li Y, Zhang H, *et al*. Effectiveness of endoscopic submucosal dissection vs gastrectomy for early gastric cancer and precancerous lesions. Journal of Third Military Medical University 2014;36:1507-1511.
78. Bao L, Gao H, Pu L, Sui C, Ji K, Wang F, *et al*. Comparison of clinical outcomes and prognosis between surgery and endoscopic submucosal dissection in patients with synchronous multifocal early gastric cancer. BMC Surg 2023;23:292.
79. Wang Z, Xia Q, Li A, Lv Q. Comparison of the effects of endoscopic submucosal dissection and laparoscopic distal radical surgery on the rehabilitation and quality of life of patients with early gastric cancer. Am J Transl Res 2023;15:2183-2190.
80. Qian M, Sheng Y, Wu M, Wang S, Zhang K. Comparison between Endoscopic Submucosal Dissection and Surgery in Patients with Early Gastric Cancer. Cancers (Basel) 2022;14:3603.
81. Hahn KY, Park CH, Lee YK, Chung H, Park JC, Shin SK, *et al*. Comparative study between endoscopic submucosal dissection and surgery in patients with early gastric cancer. Surg Endosc 2018;32:73-86.
82. Quero G, Fiorillo C, Longo F, Laterza V, Rosa F, Cina C, *et al*. Propensity score-matched comparison of short- and long-term outcomes between surgery and endoscopic submucosal dissection (ESD) for intestinal type early gastric cancer (EGC) of the middle and lower third of the stomach: a European tertiary referral center experience. Surg Endosc 2021;35:2592-2600.
83. Kang S, Lee JH, Kim Y, Park K, Na HK, Ahn JY, *et al*. Comparison of endoscopic submucosal dissection and surgery for early gastric cancer that is not indicated for endoscopic resection in elderly patients. Surg Endosc 2023;37:4766-4773.
84. Park CH, Lee H, Kim DW, Chung H, Park JC, Shin SK, *et al*. Clinical safety of endoscopic submucosal dissection compared with surgery in elderly patients with early gastric cancer: a propensity-matched analysis. Gastrointest Endosc 2014;80:599-609.
85. Fukunaga S, Nagami Y, Shiba M, Ominami M, Tanigawa T, Yamagami H, *et al*. Long-term prognosis of expanded-indication differentiated-type early gastric cancer treated with endoscopic submucosal dissection or surgery using propensity score analysis. Gastrointest Endosc 2017;85:143-152.
86. Banks M, Graham D, Jansen M, Gotoda T, Coda S, di Pietro M, *et al*. British Society of Gastroenterology guidelines on the diagnosis and management of patients at risk of gastric

adenocarcinoma. Gut 2019;68:1545-1575.
87. Park CH, Yang DH, Kim JW, Kim JH, Kim JH, Min YW, *et al*. Clinical Practice Guideline for Endoscopic Resection of Early Gastrointestinal Cancer. Clin Endosc 2020;53:142-166.
88. Pimentel-Nunes P, Libânio D, Bastiaansen BAJ, Bhandari P, Bisschops R, Bourke MJ, *et al*. Endoscopic submucosal dissection for superficial gastrointestinal lesions: European Society of Gastrointestinal Endoscopy (ESGE) Guideline - Update 2022. Endoscopy 2022;54:591-622.
89. Icaza-Chávez ME, Tanimoto MA, Huerta-Iga FM, Remes-Troche JM, Carmona-Sánchez R, Ángeles-Ángeles A, *et al*. The Mexican consensus on the detection and treatment of early gastric cancer. Rev Gastroenterol Mex (Engl Ed) 2020;85:69-85.
90. Association JGC. Japanese Gastric Cancer Treatment Guidelines 2021 (6th edition). Gastric Cancer 2023;26:1-25.
91. Bang CS, Baik GH, Shin IS, Kim JB, Suk KT, Yoon JH, *et al*. Endoscopic submucosal dissection for early gastric cancer with undifferentiated-type histology: A meta-analysis. World J Gastroenterol 2015;21:6032-6043.
92. Bang CS, Yang YJ, Lee JJ, Baik GH. Endoscopic Submucosal Dissection of Early Gastric Cancer with Mixed-Type Histology: A Systematic Review. Dig Dis Sci 2020;65:276-291.
93. Yao M, Gao H, Tao L, Wang M. Meta-analysis of the efficacy of endoscopic submucosal dissection and surgery in the treatment of undifferentiated early gastric cancer. Chin J Operat Proced Gen Surg (Electron Ed) 2023;17:46-51.
94. Ludwig K, Möller D, Bernhardt J. [Surgical management for early stage gastric cancer]. Chirurg 2018;89:347-357.
95. Zhu L, Li H, Dai J, Xue H, Gao Y, Song Y, *et al*. The pathological analysis and clinical evaluation for early gastric cancer after endoscopic submucosal dissection or surgery. Chin J Dig Endosc 2013;30:28-32.
96. Zhang Q, Zhang Y, Xue L, He S, Dou L, Liu Y, *et al*. Risk factors for endoscopic resection beyond expanded indication of undifferentiated early gastric cancer. Chin J Dig Endosc 2020;37:886-891.
97. Chen L, Chen C, Zheng Y, Lv B. Changes of endoscopic submucosal dissection indications for undifferentiated early gastric cancer. Chin J Dig Endosc 2024;41:532-536.
98. Yao H, He J, Qian Z, Zhao Z, Hu J, Shao Q. Application of Middle Gastrectomy Combined with Regional Lymph Node Dissection in Early Gastric Body Cancer. Zhejiang Med J 2016;38:1928-1930.
99. Chen Q, Li H, Chai N, Linghu E. Construction and application prospects of the cure rate evaluation system for super minimally invasive surgery in early gastric cancer. Chin J Gastrointest Endosc (Electron Ed) 2025;12:3-6.
100. Chen Q, Li H, Yuan Y, Ning B, Lyu K, Shao Q, *et al*. Prelim inary study of super minimally invasive stepwise full-thickness resection for the treatment of gastric cancer. Chin J Gastrointest Endosc (Electron Ed) 2025;12:15-18.
101. Nakamura F, Saito Y, Sakamoto T, Otake Y, Nakajima T, Yamamoto S, *et al*. Potential perioperative advantage of colorectal endoscopic submucosal dissection versus laparoscopy-assisted colectomy. Surg Endosc 2015;29:596-606.
102. Belderbos TD, van Erning FN, de Hingh IH, van Oijen MG, Lemmens VE, Siersema PD. Long-term Recurrence-free Survival After Standard Endoscopic Resection Versus Surgical Resection of Submucosal Invasive Colorectal Cancer: A Population-based Study. Clin Gastroenterol Hepatol 2017;15:403-411.e1.

103. Wang SJ, Wu ML, Zhang LW, Guo XQ, Xu ZB, Er LM, *et al*. [The value of endoscopic mucosal resection for dysplasia and early-stage cancer of the esophagus and gastric cardia]. Zhonghua Zhong Liu Za Zhi 2008;30:853-857.
104. Zhao Y, Zhang B, Mao J. Meta-analysis of the Efficacy and Safety of ESD and Surgical Treatment for Early Colorectal Cancer. Journal of Huzhou University 2019;1:67-73.
105. Tamaru Y, Oka S, Tanaka S, Nagata S, Hiraga Y, Kuwai T, *et al*. Long-term outcomes after treatment for T1 colorectal carcinoma: a multicenter retrospective cohort study of Hiroshima GI Endoscopy Research Group. J Gastroenterol 2017;52:1169-1179.
106. Zhang C. Pathological Analysis and Efficacy Evaluation of Early-Stage Colorectal Cancer After Endoscopic Resection or Surgical Treatment. Medical Diet and Health(chinese) 2018;7:85-86.
107. Guo W. Pathological Analysis and Efficacy Evaluation of Early-Stage Colorectal Cancer After Endoscopic Resection or Surgical Treatment. World Latest Medicine Information 2019;19:143-144.
108. Kiriyama S, Saito Y, Yamamoto S, Soetikno R, Matsuda T, Nakajima T, *et al*. Comparison of endoscopic submucosal dissection with laparoscopic-assisted colorectal surgery for early-stage colorectal cancer: a retrospective analysis. Endoscopy 2012;44:1024-1030.
109. Heo J, Jeon SW, Jung MK, Kim SK, Kim J, Kim S. Endoscopic resection as the first-line treatment for early colorectal cancer: comparison with surgery. Surg Endosc 2014;28:3435-3442.
110. Tribonias G, Komeda Y, Leontidis N, Anagnostopoulos G, Palatianou M, Mpellou G, *et al*. Endoscopic intermuscular dissection (EID) for removing early rectal cancers and benign fibrotic rectal lesions. Tech Coloproctol 2023;27:1393-1400.
111. Moons LMG, Bastiaansen BAJ, Richir MC, Hazen WL, Tuynman J, Elias SG, *et al*. Endoscopic intermuscular dissection for deep submucosal invasive cancer in the rectum: a new endoscopic approach. Endoscopy 2022;54:993-998.
112. Zhang L, Liu Z, Chen J, Liu Y, Ji M, Kang J, *et al*. Clinical efficacy of endoscopic mucosal resection versus endoscopic submucosal dissection for patients with colorectal cancer: a Meta-analysis. Chin J Endosc 2019;25:9-18.
113. Wang S, Gao S, Yang W, Guo S, Li Y. Endoscopic submucosal dissection versus local excision for early rectal cancer: a systematic review and meta-analysis. Tech Coloproctol 2016;20:1-9.
114. McCarty TR, Bazarbashi AN, Hathorn KE, Thompson CC, Aihara H. Endoscopic submucosal dissection (ESD) versus transanal endoscopic microsurgery (TEM) for treatment of rectal tumors: a comparative systematic review and meta-analysis. Surg Endosc 2020;34:1688-1695.
115. Lim XC, Nistala KRY, Ng CH, Lin SY, Tan DJH, Ho KY, *et al*. Endoscopic submucosal dissection vs endoscopic mucosal resection for colorectal polyps: A meta-analysis and meta-regression with single arm analysis. World J Gastroenterol 2021;27:3925-3939.
116. Gu F, Jiang W, Zhu J, Ma L, He B, Zhai H. Risk factors for unsuccessful colorectal endoscopic submucosal dissection: A systematic review and meta-analysis. Dig Liver Dis 2024;56:1288-1297.
117. Yin K, Qu J, Chen J, Xu M, Dang S, Fan X, *et al*. Clinical efficacy of laparoscopic surgery combined with colonoscopy procedure in the treatment of early colorectal tumor. Chin J Gen Pract 2018;16:1810-1812.
118. Hartwig MFS, Bulut M, Ravn-Eriksen J, Hansen LB, Bojesen RD, Klein MF, *et al*. Combined endoscopic and laparoscopic surgery (CELS) for early colon cancer in high-risk patients. Surg Endosc 2023;37:8511-8521.
119. Ferlitsch M, Hassan C, Bisschops R, Bhandari P, Dinis-Ribeiro M, Risio M, *et al*. Colorectal polypectomy and endoscopic mucosal resection: European Society of Gastrointestinal Endoscopy

(ESGE) Guideline - Update 2024. Endoscopy 2024;56:516-545.
120. Benson AB, Venook AP, Al-Hawary MM, Arain MA, Chen YJ, Ciombor KK, *et al*. Colon Cancer, Version 2.2021, NCCN Clinical Practice Guidelines in Oncology. J Natl Compr Canc Netw 2021;19:329-359.
121. Li W, Xiang XX, Da Wang H, Cai CJ, Cao YH, Liu T. Transanal endoscopic microsurgery versus radical resection for early-stage rectal cancer: a systematic review and meta- analysis. Int J Colorectal Dis 2023;38:49.
122. Chen YY, Liu ZH, Zhu K, Shi PD, Yin L. Transanal endoscopic microsurgery versus laparoscopic lower anterior resection for the treatment of T1-2 rectal cancers. Hepatogastroenterology 2013;60:727-732.
123. Saito Y, Uraoka T, Yamaguchi Y, Hotta K, Sakamoto N, Ikematsu H, *et al*. A prospective, multicenter study of 1111 colorectal endoscopic submucosal dissections (with video). Gastrointest Endosc 2010;72:1217-1225.
124. Cao Y, Liao C, Tan A, Gao Y, Mo Z, Gao F. Meta-analysis of endoscopic submucosal dissection versus endoscopic mucosal resection for tumors of the gastrointestinal tract. Endoscopy 2009;41:751-757.
125. Kuellmer A, Mueller J, Caca K, Aepli P, Albers D, Schumacher B, *et al*. Endoscopic full-thickness resection for early colorectal cancer. Gastrointest Endosc 2019;89:1180-1189.e1.
126. Ferreira MF, Marques M, Morais R, Lemmers A, Macedo G, Santos-Antunes J. Endoscopic Submucosal Dissection Is Safe and Effective for Lesions Located at the Anorectal Junction: Analysis from Two Referral European Centers. GE Port J Gastroenterol 2024;31:41-47.
127. Chen S. Retrospective Analysis of Efficacy After Endoscopic Resection for Early-Stage Colorectal Cancer. Guangdong, China: Southern Medical University; 2022.
128. Kim MN, Kang JM, Yang JI, Kim BK, Im JP, Kim SG, *et al*. Clinical features and prognosis of early colorectal cancer treated by endoscopic mucosal resection. J Gastroenterol Hepatol 2011;26:1619-1625.
129. Zwager LW, Bastiaansen BAJ, van der Spek BW, Heine DN, Schreuder RM, Perk LE, *et al*. Endoscopic full-thickness resection of T1 colorectal cancers: a retrospective analysis from a multicenter Dutch eFTR registry. Endoscopy 2022;54:475-485.
130. Park JJ, Cheon JH, Kwon JE, Shin JK, Jeon SM, Bok HJ, *et al*. Clinical outcomes and factors related to resectability and curability of EMR for early colorectal cancer. Gastrointest Endosc 2011;74:1337-1346.
131. Tanaka S, Oka S, Kaneko I, Hirata M, Mouri R, Kanao H, *et al*. Endoscopic submucosal dissection for colorectal neoplasia: possibility of standardization. Gastrointest Endosc 2007;66:100-107.
132. Lee EJ, Lee JB, Lee SH, Kim DS, Lee DH, Lee DS, *et al*. Endoscopic submucosal dissection for colorectal tumors--1,000 colorectal ESD cases: one specialized institute's experiences. Surg Endosc 2013;27:31-39.
133. Hershorn O, Ghuman A, Karimuddin AA, Raval MJ, Phang PT, Brown CJ. Local Recurrence-Free Survival After TaTME: A Canadian Institutional Experience. Dis Colon Rectum 2024;67:664-673.
134. Hotta K, Yamaguchi Y, Saito Y, Takao T, Ono H. Current opinions for endoscopic submucosal dissection for colorectal tumors from our experiences: indications, technical aspects and complications. Dig Endosc 2012;24 Suppl 1:110-116.

135. Takeuchi Y, Ohta T, Matsui F, Nagai K, Uedo N. Indication, strategy and outcomes of endoscopic submucosal dissection for colorectal neoplasm. Dig Endosc 2012;24 Suppl 1:100-104.
136. Benson AB, Venook AP, Al-Hawary MM, Azad N, Chen YJ, Ciombor KK, *et al*. Rectal Cancer, Version 2.2022, NCCN Clinical Practice Guidelines in Oncology. J Natl Compr Canc Netw 2022;20:1139-1167.
137. Department of Medical Administration NHCotPsRoC, Association. OSoCM, Gu JW, J. P. Chinese Protocol of Diagnosis and Treatment of Colorectal Cancer (2023 Edition). J Digest Oncol (Electron Vers) 2023;15:177-206.
138. Argilés G, Tabernero J, Labianca R, Hochhauser D, Salazar R, Iveson T, *et al*. Localised colon cancer: ESMO Clinical Practice Guidelines for diagnosis, treatment and follow-up. Ann Oncol 2020;31:1291-1305.
139. Hashiguchi Y, Muro K, Saito Y, Ito Y, Ajioka Y, Hamaguchi T, *et al*. Japanese Society for Cancer of the Colon and Rectum (JSCCR) guidelines 2019 for the treatment of colorectal cancer. Int J Clin Oncol 2020;25:1-42.
140. National Clinical Research Center for Digestive Diseases (Shanghai),Chinese Society of Gastrointestinal Endoscopy, Chinese Anti-Cancer Association Tumor Endoscopy Committee, Chinese Medical Doctor Association Endoscopy Physicians, Branch Gastrointestinal Endoscopy Committee, Chinese Medical Doctor Association Endoscopy Physicians, Branch Endoscopy Health Management and Physical Examination Committee, Li Z, *et al*. Expert consensus on management strategies forprecancerous lesions and conditions of colorectal cancer in China. Chin J Dig Endosc 2022;39:1–18.202. Colorectal Group DEBoCMA, Side L, Enqiang L. Expert consensus on endoscopic diagnosis and treatment for colorectal cancer and precancerous lesions in China (2023, Guangzhou). Chin J Dig Endosc 2023;40:505–520.
141. Colorectal Group DEBoCMA, Side L, Enqiang L. Expert consensus on endoscopic diagnosis and treatment for colorectal cancer and precancerous lesions in China (2023, Guangzhou). Chin J Dig Endosc 2023;40:505-520.
142. Creavin B, Ryan E, Martin ST, Hanly A, O'Connell PR, Sheahan K, *et al*. Organ preservation with local excision or active surveillance following chemoradiotherapy for rectal cancer. Br J Cancer 2017;116:169-174.
143. Rullier E, Rouanet P, Tuech JJ, Valverde A, Lelong B, Rivoire M, *et al*. Organ preservation for rectal cancer (GRECCAR 2): a prospective, randomised, open-label, multicentre, phase 3 trial. Lancet 2017;390:469-479.
144. Stijns RCH, de Graaf EJR, Punt CJA, Nagtegaal ID, Nuyttens J, van Meerten E, *et al*. Long-term Oncological and Functional Outcomes of Chemoradiotherapy Followed by Organ-Sparing Transanal Endoscopic Microsurgery for Distal Rectal Cancer: The CARTS Study. JAMA Surg 2019;154:47-54.
145. Zhao GH, Deng L, Ye DM, Wang WH, Yan Y, Yu T. Efficacy and safety of wait and see strategy versus radical surgery and local excision for rectal cancer with cCR response after neoadjuvant chemoradiotherapy: a meta-analysis. World J Surg Oncol 2020;18:232.
146. Huang Y, Huang S, Chi P, Wang X, Lin H, Lu X, *et al*. Rectum-preserving surgery after consolidation neoadjuvant therapy or totally neoadjuvant therapy for low rectal cancer: a preliminary report. Chin J Gastrointest Surg 2020;23:281-288.
147. Xue X, Zhou J, Lin G, Bai XS, Xiao Y, Wu B, *et al*. An initial exploration of the application of transanal endoscopic microsurgery in rectal cancer patients with clinical complete response after

neoadjuvant chemoradiotherapy. Chin J Gastrointest Surg 2019;22:560-565.
148. Kundel Y, Brenner R, Purim O, Peled N, Idelevich E, Fenig E, *et al*. Is local excision after complete pathological response to neoadjuvant chemoradiation for rectal cancer an acceptable treatment option? Dis Colon Rectum 2010;53:1624-1631.
149. Qiu X, Zhou J, Lin G, Lu J, Niu B, Qie H. Application of local resection in patients with mid-to-low rectal cancer achieving clinical complete or near-complete response after neoadjuvant chemoradiotherapy. Chin J Gen Surg 2025;34:760-768.
150. Expert Group on Early Diagnosis and Treatment of Cancer CSoO, Chinese Medical Association, Kefeng D, Zhongfa X, Min D. Expert consensus on the early diagnosis and treatment of colorectal cancer in China (2023 edition). Chin J Gen Surg (Electron Ed) 2024;18:1-13.
151. Glynne-Jones R, Wyrwicz L, Tiret E, Brown G, Rödel C, Cervantes A, *et al*. Rectal cancer: ESMO Clinical Practice Guidelines for diagnosis, treatment and follow-up. Ann Oncol 2017;28:iv22-iv40.
152. (CWWD) CWWDRCG, Surgery CSoC, Association CM, Committee CCPS, Committee CC, Aiwen W, *et al*. Chinese expert consensus on the watch and wait strategy in rectal cancer patients after neoadjuvant treatment (2024 version). Chin J Gastrointest Surg 2024;27:301-315.
153. Dattani M, Heald RJ, Goussous G, Broadhurst J, São Julião GP, Habr-Gama A, *et al*. Oncological and Survival Outcomes in Watch and Wait Patients With a Clinical Complete Response After Neoadjuvant Chemoradiotherapy for Rectal Cancer: A Systematic Review and Pooled Analysis. Ann Surg 2018;268:955-967.
154. Dossa F, Chesney TR, Acuna SA, Baxter NN. A watch-and-wait approach for locally advanced rectal cancer after a clinical complete response following neoadjuvant chemoradiation: a systematic review and meta-analysis. Lancet Gastroenterol Hepatol 2017;2:501-513.
155. Smith JJ, Strombom P, Chow OS, Roxburgh CS, Lynn P, Eaton A, *et al*. Assessment of a Watch-and-Wait Strategy for Rectal Cancer in Patients With a Complete Response After Neoadjuvant Therapy. JAMA Oncol 2019;5:e185896.
156. Spadaccini M, Fugazza A, Frazzoni L, Leo MD, Auriemma F, Carrara S, *et al*. Endoscopic papillectomy for neoplastic ampullary lesions: A systematic review with pooled analysis. United European Gastroenterol J 2020;8:44-51.
157. Hollenbach M, Heise C, Abou-Ali E, Gulla A, Auriemma F, Soares K, *et al*. Endoscopic papillectomy versus surgical ampullectomy for adenomas and early cancers of the papilla: a retrospective Pancreas2000/European Pancreatic Club analysis. Gut 2025;74:397-409.
158. Garg R, Thind K, Bhalla J, Simonson MT, Simons-Linares CR, Singh A, *et al*. Long-term recurrence after endoscopic versus surgical ampullectomy of sporadic ampullary adenomas: a systematic review and meta-analysis. Surg Endosc 2023;37:5022-5044.
159. Vanbiervliet G, Strijker M, Arvanitakis M, Aelvoet A, Arnelo U, Beyna T, *et al*. Endoscopic management of ampullary tumors: European Society of Gastrointestinal Endoscopy (ESGE) Guideline. Endoscopy 2021;53:429-448.
160. Fritzsche JA, Fockens P, Barthet M, Bruno MJ, Carr-Locke DL, Costamagna G, *et al*. Expert consensus on endoscopic papillectomy using a Delphi process. Gastrointest Endosc 2021;94:760-773.e18.
161. Itoi T, Ryozawa S, Katanuma A, Kawashima H, Iwasaki E, Hashimoto S, *et al*. Clinical practice guidelines for endoscopic papillectomy. Dig Endosc 2022;34:394-411.
